# Regulatory role of *tetR* gene in a novel gene cluster of * Acidovorax avenae* subsp. *avenae* RS-1 under oxidative stress

**DOI:** 10.3389/fmicb.2014.00547

**Published:** 2014-10-21

**Authors:** He Liu, Chun-Lan Yang, Meng-Yu Ge, Muhammad Ibrahim, Bin Li, Wen-Jun Zhao, Gong-You Chen, Bo Zhu, Guan-Lin Xie

**Affiliations:** ^1^State Key Laboratory of Rice Biology, Institute of Biotechnology, Zhejiang UniversityHangzhou, China; ^2^Department of Plant Pathology, University of California DavisDavis, CA, USA; ^3^Department of Biosciences, COMSATS Institute of Information TechnologySahiwal, Pakistan; ^4^Chinese Academy of Inspection and QuarantineBeijing, China; ^5^School of Agriculture and Biology, Shanghai Jiao Tong UniversityShanghai, China

**Keywords:** horizontal gene transfer, *tetR*, repressor, oxidative stress, EMSA, proteome profiling

## Abstract

*Acidovorax avenae* subsp. *avenae* is the causal agent of bacterial brown stripe disease in rice. In this study, we characterized a novel horizontal transfer of a gene cluster, including *tetR*, on the chromosome of *A. avenae* subsp. *avenae* RS-1 by genome-wide analysis. TetR acted as a repressor in this gene cluster and the oxidative stress resistance was enhanced in *tetR*-deletion mutant strain. Electrophoretic mobility shift assay demonstrated that TetR regulator bound directly to the promoter of this gene cluster. Consistently, the results of quantitative real-time PCR also showed alterations in expression of associated genes. Moreover, the proteins affected by TetR under oxidative stress were revealed by comparing proteomic profiles of wild-type and mutant strains via 1D SDS-PAGE and LC-MS/MS analyses. Taken together, our results demonstrated that *tetR* gene in this novel gene cluster contributed to cell survival under oxidative stress, and TetR protein played an important regulatory role in growth kinetics, biofilm-forming capability, superoxide dismutase and catalase activity, and oxide detoxicating ability.

## INTRODUCTION

*Acidovorax avenae* subsp. *avenae*, (formerly *Pseudomonas avenae*) is a Gram-negative, rod-shaped causal bacterial agent, which infects many economically important crops, including rice, maize, millet, oats, sugarcane, and foxtail (Song et al., 2004). In rice, it causes bacterial brown stripe (BBS) disease, which is a devastating rice disease in some areas in Asia, Africa, North America, and Europe (Xie et al., 1998, 2011). Although this worldwide rice disease has caused significant yield and quality losses, currently little is known about this bacterial pathogen, especially at the molecular and cellular levels. Recently, the draft genome sequence of *A. avenae* subsp. *avenae* RS-1 has been released (Xie et al., 2011). This opens a new avenue to study this pathogen that will ultimately lead to better control of this disease.

Comparative genomic analysis with closely related species has revealed several novel horizontally transferred gene clusters in this pathogen (Xie et al., 2011). Horizontal gene transfer (HGT) is a progress in which an organism incorporates genetic material from other organisms without vertical inheritance. This progress has been considered as a major driving force in bacterial evolution (Ochman et al., 2000). HGT can transfer not only one gene to the recipient that enhances the niche adaption, but even an entire gene cluster that may dramatically alter the metabolic repertoire of the host, even creating functional novelties, including stress response (Slade and Radman, 2011).

Bacterial cells are always exposed to various adverse conditions and surroundings, which compel them to adapt to conditions far from optimum. For example, oxidative stress occurs when reactive oxygen species are naturally generated in aerobically growing cells where incomplete reduction of molecular oxygen happens during their normal metabolism. These toxic compounds induce oxidative stress to the cell, damaging nucleic acids, and proteins (Imlay, 2013). The reactive oxygen species include hydrogen peroxide (H_2_O_2_), superoxide radical (O_2_⋅), and hydroxyl radical (HO⋅). Reactive oxygen species can also occur from a variety of environmental sources, such as redox-cycling agents and ionizing radiation (Sies, 1997). Paraquat, one kind of redox-cycling agents, can generate endogenous superoxide stress and react with respiratory chain components in bacterial cells (Tu et al., 2012). In most cases, survival in this unstable environment requires a wide range of adaptive, effective feedbacks mediated or triggered by regulatory proteins (Ramos et al., 2005). The TetR protein family is a common class of transcriptional regulator found in a diversity of bacteria. It has been reported that this protein family can take function in various physiological processes, such as biosynthesis of antibiotics and expression of enzymes implicated in catabolic pathways, and multidrug resistance (MDR; August et al., 1998; van der Geize et al., 2000; Cho et al., 2003; Rand et al., 2006). However, only about 5% among them have been fully characterized (Ramos et al., 2005; Hillerich and Westpheling, 2008), and fewer were reported in phytopathogens (Ramos et al., 2005).

Whole genome sequencing of the *A. avenae* subsp. *avenae* RS-1 strain has provided a general view of HGT, which may have enhanced its pathogenicity or survival ability. In this study, we found a horizontally transferred gene cluster containing four genes including the *tetR* gene and paraquat-inducible genes (*pqiAA’B*). We investigated the role of TetR in regulating expression of this gene cluster and found it negatively regulated its expression. We also found TetR plays a role in cell growth rate, biofilm formation, and oxidative stress survival.

## MATERIALS AND METHODS

### BACTERIA STRAINS, PLASMIDS, AND CULTURE CONDITIONS

Strains and plasmids used in this study were listed in **Table 1**. *A. avenae* subsp. *avenae* strains were grown in Luria-Bertani (LB) broth or LB with 1.5% (w/v) agar at 30°C. LB agar without sucrose and LB agar containing 10% (w/v) sucrose were used for deletion mutagenesis (Li et al., 2011). The bacterial optical density (OD600) was determined with a spectrophotometer (Perkin Elmer Lambda35 UV/VIS). When required, antibiotics were added at the following concentrations: ampicillin (Amp), 100 μg ml^-1^; chloromycetin (Chl), 3.4 μg ml^-1^; kanamycin (Km), 50 μg ml^-1^; and rifampicin (Rif), 100 μg ml^-1^.

**Table 1:** Strains and plasmids used in this study.

Strain or plasmid	Relevant characteristics^a^	Source or reference
**Strains**
***Acidovorax avenae* subsp. *avenae***
RS-1	Rif^R^; The pathogen of bacterial brown stripe of rice, isolated from the diseased rice from Zhejiang province in China	Lab collection
RS-*tetR*	Rif^R^, RS-1 in-frame deletion mutation defective in *tetR*	This study
RS-*tetR*-comp	Rif^R^, Amp^R^, Chl^R^; RS-*tetR* complemented with pRADK-*tetR*	This study
***Escherichia coli***
DH5α	F^-^ Φ80d *lacZ*ΔM15Δ(*lacZYA*-*argF*) U169 *recA1 endA1, hsdR17*(r_k_^-^, m_k_^+^) *phoA supE44λ^-^ thi-1 gyrA96 relA1*	This study
S17-1(λ pir)	λ Lysogenic S17-1 derivative producing π protein for replication of plasmids carrying *oriR6K*; *recAprohsdR*RP4-2-Tc::Mu-Km::Tn7 λ^-^ pir	Simon et al. (1983)
BL21(DE3)	F^-^ *ompT* *hsdS20*(r_b_^-^, m_b_^-^) *gal*	Novagen
**Plasmids**
pGEM-T	Amp^R^; cloning vector	Promega
pKMS1	Km^R^; R6K-based suicide vector; requires the *pir*-encoded π protein for replication	Li et al. (2011), Zou et al. (2011)
pRADK	Amp^R^, Chl^R^; broad host expression vector	Gao et al. (2005)
pGEM-T-*tetR*	Amp^R^; pGEM-T with a 726 bp fragment of *tetR* (including the entire open reading frame) from strain RS-1	This study
pKMS-*tetR*	Km^R^; pKMS1 containing the *tetR*1 (250 bp) and *tetR*2 (186 bp) DNA fragment of gene *tetR* from strain RS-1; used to create mutant RS-*tetR*	This study
pRADK-*tetR*	Amp^R^, Chl^R^; pRADK plasmid containing the *tetR* gene from strain RS-1, excised from pGEM-T-*tetR*; utilize to complement	This study
pGEX6P-1	pBR322 origin, *lacI*, GST-tag, Km^R^, expression vector	Novagen
pGEX6P-*tetR*	pGEX6P-1 expression TetR, GST-tagged, Km^R^	This study

*^a^Amp^R^, Chl ^R^, Km^R^, Rif ^R^, indicate ampicillin-, chloromycetin-, kanamycin-, rifampicin-resistant, respectively.*

### PLANT MATERIAL AND INOCULATION FOR BACTERIAL VIRULENCE ASSAY

The cultivar of rice plant used in this study was ZheYou#1 (susceptible to *A. avenae* subsp. *avenae*). Experiments were performed in greenhouse. For inoculating plants, strains were grown in LB broth for 48 h, diluted in ddH_2_O, and adjusted to OD600 of 0.6 (1 × 10^8^ CFU ml^-1^; Liu et al., 2012). Leaf-clip inoculation was carried out on 6 weeks-old rice plants. Lesion length was measured at 14 days post-inoculation (Yu et al., 2011).

### PHYLOGENETIC ANALYSIS

Each gene of interest was compared against sequences in the NCBI nr database using BLASTp (Altschul et al., 1997) followed by extracting the sequence of each specie with highest similarity for further study. We considered two genes as homologs when E-value <10^-5^ and when the alignment similarity was higher than 40% with more than 80% coverage (Nogueira et al., 2009). ClustalW was used for sequences alignment (Thompson et al., 1994), and the conserved region of each alignment was trimmed with Gblocks (Castresana, 2000) under stringent settings described previously (Ciccarelli et al., 2006). Maximum Likelihood (Fang et al., 1999) phylogenies were evaluated and built by PhyML (Guindon and Gascuel, 2003) using a JTT model and a gamma distribution with eight rate categories. We performed 1,000 bootstraps to gain branch support values.

### CONSTRUCTION OF *tetR-*DEFECTIVE DELETION MUTANT AND COMPLEMENTATION

To investigate the role of *tetR* in *A. avenae* subsp. *avenae*, an in-frame deletion mutation by double cross-over events of this gene (RS_1091) was constructed as described previously using the suicide vector pKMS1 (Li et al., 2011; Zou et al., 2011) by homologous recombination on the background of wild-type strain RS-1. Briefly, two fragments flanking the start and stop codons of target gene, *tetR*1 (250 bp) and *tetR*2 (186 bp), were amplified from the wide-type genomic DNA (gDNA) with primer pairs *tetR*1-F/*tetR*1-R and *tetR*2-F/*tetR*2-R (Table S1), respectively. The two fragments were digested with BamHI and HindIII and ligated into the vector pKMS1 at the corresponding sites, resulting in pKMS-*tetR*. This recombinant plasmid was transferred into *Escherichia coli* S17-1 λ pir (Simon et al., 1983) and then introduced into *A. avenae* subsp. *avenae* by filter mating (Smith and Guild, 1980). The single colonies that emerged on LB plates containing kanamycin and rifampicin at 30°C for 2 days were then transferred to LB broth medium followed by incubating at 30°C and 200 rpm for 16 h. Afterward, the bacterial culture was plated to LB agar containing 10% (w/v) sucrose for the second cross-over through *sacB* and sucrose-positive selection. After sucrose resistant colonies were patched onto LB and LB plus kanamycin plates, respectively, individual colony that grew normally on LB plates, but did not grow on LB containing kanamycin, was considered potential deletion mutants where double exchange homologous recombination events occurred at the two *tetR* fragments, resulting in a 267 bp deletion. One of the mutants, named RS-*tetR*, was subsequently verified by PCR and sequencing before using for further study.

In order to complement the RS-*tetR* strain, the 1062 bp fragment containing full-length of *tetR* gene and 300 bp of its upstream region was amplified by PCR. The primers were designed based on RS-1 genome and listed in Table S1. The PCR product was cloned into pGEM-T Easy vector, verified by sequencing, and then cloned into pRADK (Gao et al., 2005). Complementation vector was introduced into mutant cells by electroporation and complementary strain was selected by resistance to Chl and Amp.

### GROWTH CURVE AND SURVIVAL ASSAYS

In order to monitor the growth rate, the growth curve assays were performed as described by Tu et al. (2012) with minor modification. 1 ml overnight culture, which was grown at 30°C with 200 rpm agitation in LB broth, was added into 99 ml fresh LB broth to OD600 of 0.05. The concentration of commercial 30% H_2_O_2_ (10 mol L^-1^) solution and 100 mmol L^-1^ paraquat (N,N′-dimethyl-4,4′-bipyridinium dichloride, C_12_H_14_Cl_2_N_2_, 257.16 *g* mol^-1^) solution (2.57 *g* solid paraquat diluted with 100 ml ddH_2_O) were used. The reactive oxygen compound (H_2_O_2_ or paraquat) was added at mid-log phase (OD600=0.3) to the indicated final concentrations. After comparing with the untreated bacterial growth curve, the sensitivity and tolerance of cells to the reagents was determined. This experiment was repeated three times independently.

### PROTEIN PURIFICATION AND LC-MS/MS ANALYSIS

Protein extraction was performed as described by Tu et al. (2012) with some modifications. Briefly, 2 ml overnight grown cells were added to 100 ml fresh LB broth to continue growing at 30°C with 200 rpm agitation. Paraquat or H_2_O_2_ was added to cells at mid-log phase (OD600=0.3) to the indicated concentration that perturbed the growth without causing cell death according to the survival assay (40/60 mM H_2_O_2_ or 0.4/0.8 mM paraquat, RS-1/RS-*tetR*). Cells were harvested by centrifugation (5,000 *g*, 10 min, 4°C) after exposure to the oxidative compounds for 60 min. Then the cells were treated with lysozyme (1 mg/mL) in the phosphate buffer (pH 8.0), disrupted by ultrasound, and centrifuged at 12,000 *g* for 15 min at 4°C in a Sorvall centrifuge to remove cell debris. A second-time centrifugation step (20,000 *g*, 30 min, 4°C) was required by by Sorvall centrifuge (Sorvall WX100, Thermo Scientific, USA). The protein content was quantified using the enhanced BCA Protein Assay Kit (Beyotime, China).

Protein extracts were subject to 1D SDS-PAGE gel composed of 5% acrylamide for stacking gel and 10% for running gel (Schägger and Von Jagow, 1987) with a mini-gel apparatus (VE-180 vertical electrophoresis bath, Tanon, China). Subsequently, the separated protein bands in the SDS-PAGE gel were visualized by silver staining.

The peptides released from trypsin digestion for LC-MS/MS analyzing were prepared in two biological replicates. LC was performed using a Dionex Ultimate 3000 nano-LC system. Firstly, the tryptic peptides were acidified by 2% acetonitrile with 0.025% trifluoroacetic acid before loading onto the Dionex Acclaim PepMap 100, C18 trap column (20 mm × 100 μm, 5 μm, 100 Å) at the flow rate of 10 μl min^-1^. Then, the Dionex Acclaim PepMap 100, C18 analytical column (150 mm × 75 μm, 3 μm, 100 Å) were applied to divide the enriched tryptic peptides by gradient elution. The Bruker amaZon electron transfer reaction (ETD) ion trap system coupled with nano source expanded the capability to identify the trapped tryptic peptides under 300–1400 m/z and 50–2200 m/z scan range for MS and MS/MS, respectively.

The MASCOT LC-MS/MS ion search algorithm (Matrix Sciences) was applied to evaluate the LC/MS spectra and sequence similarity of resulting peptides to *A. avenae* subsp. *avenae* RS-1 was compared with *Acidovorax* species accessible on NCBI. To further identify the proteins, the cross correlation scores (X corr; Lin et al., 2007) of singly-, doubly- and triply-charged peptides were fixed greater than 1.8, 2.5 and 3.5, respectively. Then, a list of peptide sequences with the highest X corr values was identified. The hydrophobic nature of proteins was accessed through evaluating the grand average of hydropathicity (GRAVY) score of peptides by ProtParam ExPASy. Furthermore, proteins were annotated by RAST automatic pipeline to analyze their function.

### ENZYME ASSAYS FOR SOD AND CATALASE

After protein purification as mentioned above, 1–2.5 μg protein was used to measure superoxide dismutase (SOD) or catalase activities. Precisely, SOD activity was determined with a SOD assay Kit-WST (Beyotime, China). The Catalase Assay Kit (Beyotime, China) was used to detect catalase activity. Briefly, for SOD activity assay, the samples were treated by WST-8 [2-(2-methoxy-4-nitrophenyl)-3-(4-nitrophenyl)-5-(2,4-disulfophenyl)- 2H-tetrazolium, monosodium salt] and incubated at 37°C for 30 min. Afterward, the absorption maximum at 450 nm was measured by Thermo Multiskan EX Micro plate Photometer (Thermo Fisher Scientific, Waltham, MA, USA). SOD activity was calculated by standard curve according to the instruction of manufacturer in kit. For catalase assay, the samples were treated with 10 μl of 250 mM H_2_O_2_ and incubated 5 min at room temperature, and the remaining H_2_O_2_ (not decomposed by catalase) was coupled with a substrate to generate *N*-4-antipyryl-3-chloro-5-sulfonate-*p*-benzoquinonemonoimine, which has an absorption maximum at 520 nm and was quantified spectrophotometrically. Catalase activity was then calculated by standard curve according to the instruction of manufacturer in kit. These experiments were repeated three times independently.

### BIOFILM FORMATION ASSAY

Biofilm formation assays were performed using the crystal violet (CV) assay method (Peeters et al., 2008). Bacteria were grown overnight with approximately 200 rpm agitation and diluted at 1:50 into fresh LB media. 100 μl of 10^8^ CFU ml^-1^ mutant or wild-type bacterial suspension was added to one well of 96-well microtitre plate with sterile ddH_2_O serving as blanks. Cells were allowed to form biofilm at 30°C for 48 h without agitation. After the planktonic organisms were removed and each well in the plate was rinsed and air-dried, 125 μl of 0.1% (w/v) CV solution was used to stain biofilm and 150 μl of 33% acetic acid was added to release the bond CV. The optical density was measured at 590 nm using a Thermo Multiskan EX Micro plate Photometer (Thermo Fisher Scientific, Waltham, MA, USA). This experiment was repeated three times independently with 12 replicates each.

### RNA EXTRACTION AND REAL-TIME PCR

The bacterial culture (OD600=1.0) was utilized for RNA extraction after exposure to the oxidative stressor (40/60 mM H_2_O_2_ or 0.4/0.8 mM paraquat, RS-1/RS-*tetR*) for 10 min. Bacterial total RNA was extracted from RS-1 and RS-*tetR* strain respectively as described by the manual of RNeasy Protect Bacteria Mini Kit (QIAGEN) and then used for generating first strand complementary DNA (cDNA) as described in the protocol of the Takara PrimeScript RT reagent Kit with gDNA Eraser (Takara). Briefly, 1 ml of bacterial cells were mixed with 2 ml of RNAprotect Bacteria Reagent before incubating for 5 min at room temperature. A pellet was obtained after centrifugation and was then treated by TE buffer (10 mM Tris⋅Cl, 1 mM EDTA; pH 8.0) containing 1 mg/ml lysozyme at room temperature for 5 min. After detecting the RNA quantity by agarose gel electrophoresis and the quality by Nanodrop ND1000 spectrophotometer V 3.5.2 (NanoDrop Technologies, Wilmington, DE, USA), 1 μg of the resulting total RNA was used at 42°C for 2 min to eliminate gDNA by gDNA Eraser and buffer before obtaining cDNA. Then the reverse transcription reaction was accomplished by incubating at 37°C for 15 min and then 85°C for 5 s in the presence of random RT primers. An ABI PRISM 7500 Real-Time PCR System and SYBR green fluorescence chemistry (SYBR Green I Master Kit, Roche Diagnostics) were employed to perform quantitative real-time RT-PCR amplification and analysis. Real-time PCR amplification was generated with 30 s of initial denaturation at 95°C, followed by 40 cycles of 5 s at 95°C, 30 s at 60°C for amplification. The reference genes (16S rRNA of both RS-1 and RS-*tetR*) were used to normalize the measurements between samples. Data analyses were based on change-in-cycling-threshold method (2^-ΔΔCt^; Livak and Schmittgen, 2001). RNA extraction was carried out with two replicates per strain and qPCR was performed with three replicates.

### EXPRESSION AND PURIFICATION OF THE tetR PROTEIN

The *tetR* gene was PCR-amplified from *A. avenae* subsp. *avenae* RS-1 genome with primers that introduced a BamHI site overlapping the translation initiation codon and a SalI site downstream of the stop codon, respectively (Table S1). A 763 bp *tetR*-containing DNA fragment was cut with BamHI and SalI and subcloned into the pGEX6P-1 expression vector (Novagen, USA) with the same restriction enzymes, yielding pGEX6P-*tetR*. Subsequently, pGEX6P-*tetR* was transformed into *E. coli* BL21(DE3) and was grown in LB medium containing 100 μg ml^-1^ ampicillin at 30°C to OD600=0.5. Afterward, the culture was grown for an additional 3 h after isopropylthiogalactoside (IPTG) was added to a final concentration of 1 mM. The cells were then harvested by centrifugation at 8,000 *g*, 4°C for 10 min, and the pellet was resuspended in 10 ml of lysis buffer (70 mM HEPES, 20 mM imidazole, 650 mM NaCl, 0.5 mM β-mercaptoethanol, 10% glycerol, pH 8; Balhana et al., 2013). Cells were disrupted on ice by ultrasonic treatment for 20 min with 8 s rest period every 16 s and the supernatant was recovered by removing the cellular debris through centrifugation (12,000 *g*, 20 min, 4°C). Subsequently, TetR protein with GST-tag was separated and eluted by GST-Tag Bind resin (Sangon Biotech, China) followed by the manufacturer and the purity was confirmed by SDS-PAGE. The concentration of the purified protein was determined using the enhanced BCA Protein Assay Kit (Beyotime, China).

### ELECTROPHORETIC MOBILITY SHIFT ASSAYS (EMSA)

A 292 bp fragment comprising the intergenic region between *tetR* and *pqiA’* was PCR amplified from *A. avenae* subsp. *avenae* RS-1 gDNA and the 3′-end of the fragment was labeled by biotin to be used as probe, according to the procedures of electrophoretic mobility shift assay (EMSA) Probe Biotin Labeling Kit (Beyotime, China). A 150 bp DNA fragment from the downstream of *pqiB* gene was worked as non-specific DNA control. Binding reactions were carried out by incubating varying concentrations of protein with 0.05 pmol of labeled probe in a total reaction volume of 20 μl. The EMSA were performed using a Light-Shift chemiluminescence EMSA kit (Beyotime, China) following the manufacturer’s instructions and the membrane was washed and detected following chemiluminescent method using Streptavidin-HRP conjugates and BeyoECL Plus reagents. ECL signals were captured and visualized by exposing the membrane to Hyperfilm^TM^ (GE Healthcare).

### STATISTICAL ANALYSES

When needed, the data of quantitative assays were analyzed by SPSS 16.0 (SPSS Inc., Chicago, IL, USA) using an analysis of variance test and the mean values were compared with the least significant differences test.

Clusters of Orthologous Groups (COGs) enrichment analysis was carried out based on the Hypergeometric Test,

$$P=\sum_{i=x}^{n}\frac{\left(\underset{i}{M}\right)\,\left(\underset{n-i}{N-M}\right)}{\left(\underset{n}{N}\right)}$$

In which, *N* means the number of genes in the RS-1 genome, *M* means the number of genes filling into one COG category in the RS-1 genome, *n* means the number of differential proteins in *tetR* mutant LC-MS/MS result and *I* means the number of genes filling into one COG category in *tetR* mutant LC-MS/MS result. *P* < 0.01 was used as the cutoff to define the significance.

## RESULTS

### THE SYNTENY AND ORIGIN OF GENE CLUSTER

**FIGURE 1:**
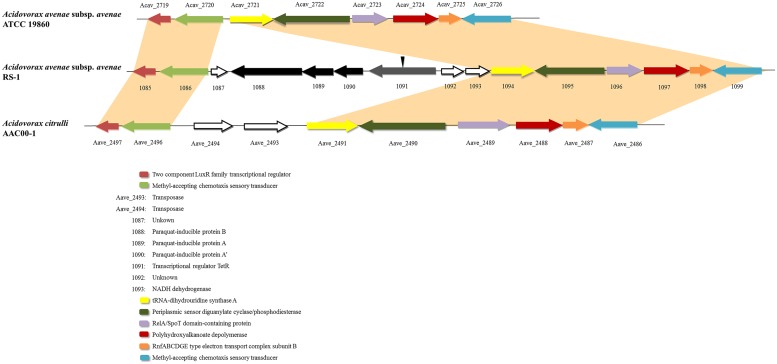
**Alignment of *Acidovorax avenae* subsp. *avenae* RS-1, ATCC 19860 and *A. citrulli* AAC00-1 chromosomes.** One cluster was found in RS-1 containing four genes including *tetR* and *pqiAA’B*. Sequences from this cluster with upstream and downstream sequences were aligned with *A. avenae* subsp. *avenae* ATCC 19860 and *A. citrulli* AAC00-1. The single-headed arrows represent the position and orientation of the gene in genome. The deleted site of the *tetR* gene was shown by inverted triangle.

Horizontal gene transfer is an important mechanism driving the evolution of microbial genomes (Gogarten and Townsend, 2005). By comparing with *A. avenae* subsp. *avenae* ATCC 19860 and *A. citrulli* AAC00-1, two most closely related genomes deposited in public database, we found that the upstream and downstream regions of this cluster were highly conserved (**Figure 1**), indicating a novel gene cluster in RS-1. This cluster encodes four genes including *pqiAA’B* and *tetR* gene. The *tetR* gene is located upstream from the *p*ara*q*uat-*i*nducible genes (*pqiAA’B*) in tandem. In the phylogenetic trees generated using the four genes in this cluster, RS-1 always grouped together with different bacterial species, with *Pseudomonas* as the out-group (Figure S1). These gene trees were highly incongruent with the bacterial species tree. Based on these results, we hypothesize that this gene cluster may be horizontally transferred from a *Pseudomonas* ancestor.

In previous reports, TetR family members are particularly abundant in microbes and play important roles in bacterial adaptability or fitness when they are exposed to adverse environmental challenges, such as oxidative stress conditions (Ramos et al., 2005). Thus, in order to understand the role of the TetR protein in RS-1, we decided to investigate its molecular function by deleting *tetR* gene in RS-1 strain (Figure S2).

### THE *tetR* MUTANT SHOWED DECREASED BACTERIAL GROWTH RATE AND INCREASED TOLERANCE TO OXIDATIVE STRESS

The growth rate of the *tetR* mutant strain RS-*tetR* decreased significantly compared with the wild-type as well as the complemented strain RS-*tetR*-comp (Figure S3). Wild-type and RS-*tetR*-comp strains entered the log phase 1 h after the culture started and the OD600 reached up to 2.2 while the RS-*tetR* strain entered the log phase much later and its OD600 only reached 1.7.

**FIGURE 2:**
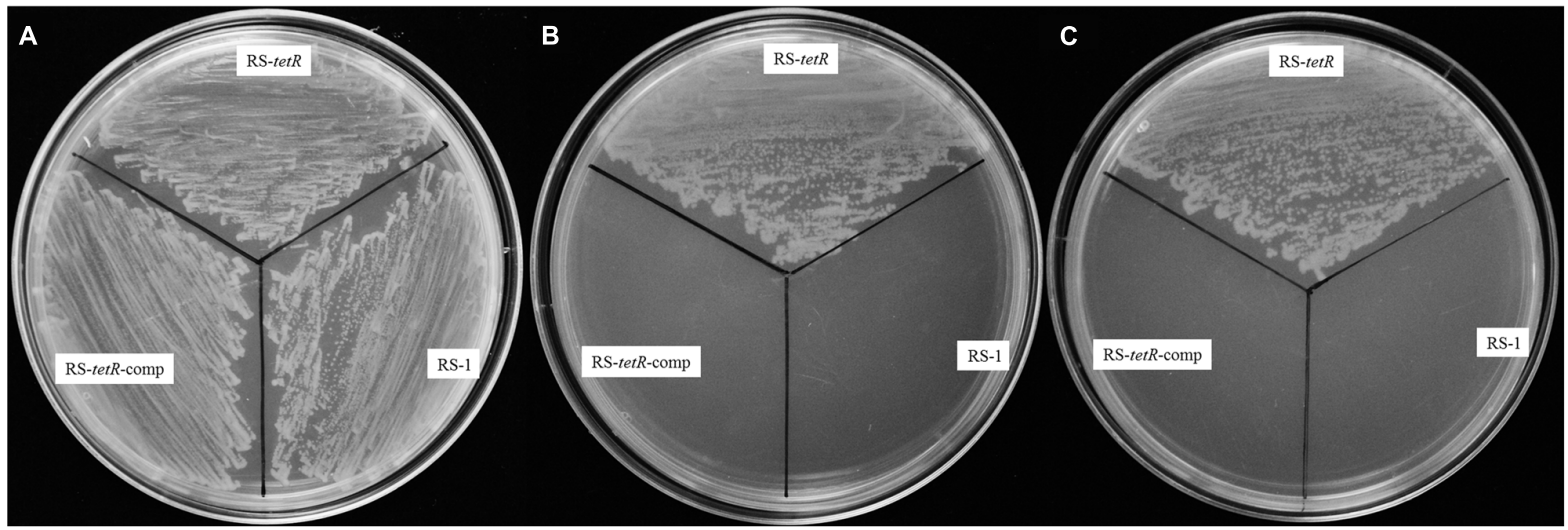
**Survival assays of RS-1, RS-*tetR* and RS-*tetR*-comp, respectively.** The mutant strain grew well on the LB plates containing paraquat or H_2_O_2_, while the growth of wild-type and complementary cells were inhibited. **(A)** Growth on LB plate without oxidative reagent; **(B,C)** Growth on LB plate with 0.8 mM paraquat and 60 mM H_2_O_2_, respectively.

In order to investigate the tolerance capability of *A. avenae* subsp. *avenae* strains to oxidative stress, we carried out a comparative analysis of the growth kinetics under different concentrations of paraquat or H_2_O_2_. Generally, paraquat or H_2_O_2_ strongly inhibited the growth of both mutant and wild-type strains. Importantly, the mutant strain showed higher resistance than wild-type strain to these reactive oxygen reagents (H_2_O_2_ or paraquat) when added at mid-log phase (OD600=0.3; as the arrow shown in Figure S4). Six different concentrations of paraquat (0.0–1.0 mM) or H_2_O_2_ (0–100 mM) were used to test their effects. At 0.4 mM paraquat or 40 mM H_2_O_2_, RS-1 and RS-*tetR*-comp were completely inhibited, showing no further growth (Figures S4A,B,E,F), whereas the RS-*tetR* was little affected and continued to grow. At 0.8 mM of paraquat or 60 mM of H_2_O_2_, growth of RS-*tetR* strain was inhibited (Figures S4C,D), whereas RS-1 and RS-*tetR*-comp cells started to die. Moreover, the results from LB plate assay were consistent with those of growth curves. The RS-*tetR* grew well on LB plates containing 0.8 mM paraquat or 60 mM H_2_O_2_, while RS-1 and RS-*tetR*-comp was inhibited (**Figure 2**). These data suggest that TetR is involved in negative regulation of cell growth and adaption under oxidative stress.

### DIFFERENCE IN BIOFILM FORMATION, BUT NO DIFFERENCE IN BACTERIAL VIRULENCE

**FIGURE 3:**
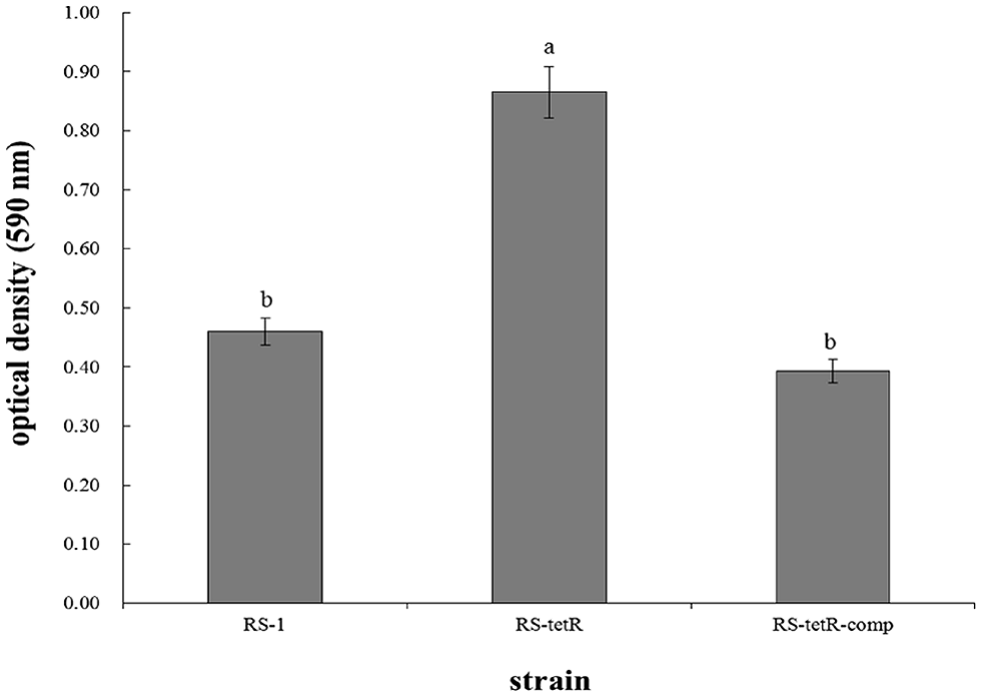
**Biofilm quantification and optical density measurements at 590 nm.** Bacterial cells were placed in 96-well plate at 30°C without agitation. After 48 h, biofilm was formed and stained by 0.1% (w/v) crystal violet solution before releasing by 33% acetic acid. Means ± SEM are shown; *n* = 12. Different letters indicated significant differences (*P* < 0.05) among treatments.

The morphological appearance and thickness of biofilm showed obvious differences after 48 h of adhesion at 30°C without agitation. Particularly, RS-*tetR* strain formed a thicker biofilm compared with RS-1 and RS-*tetR*-comp strains. When quantified, RS-*tetR* yielded readings twice as high as RS-1 and RS-*tetR*-comp (**Figure 3**). The quantitative data confirmed that the mutant strain exhibited significantly (*P* < 0.05) higher biofilm-forming capability compared to the other two strains. However, the virulence did not show significant difference comparing among wild-type, mutant and complementation strains by observation of symptoms and analysis of lesion lengths 14 days post-inoculation (Figure S5).

### INCREASED ACTIVITIES OF SOD AND CATALASE IN *tetR* MUTANT CELLS WITH OXIDATIVE STRESS

**FIGURE 4:**
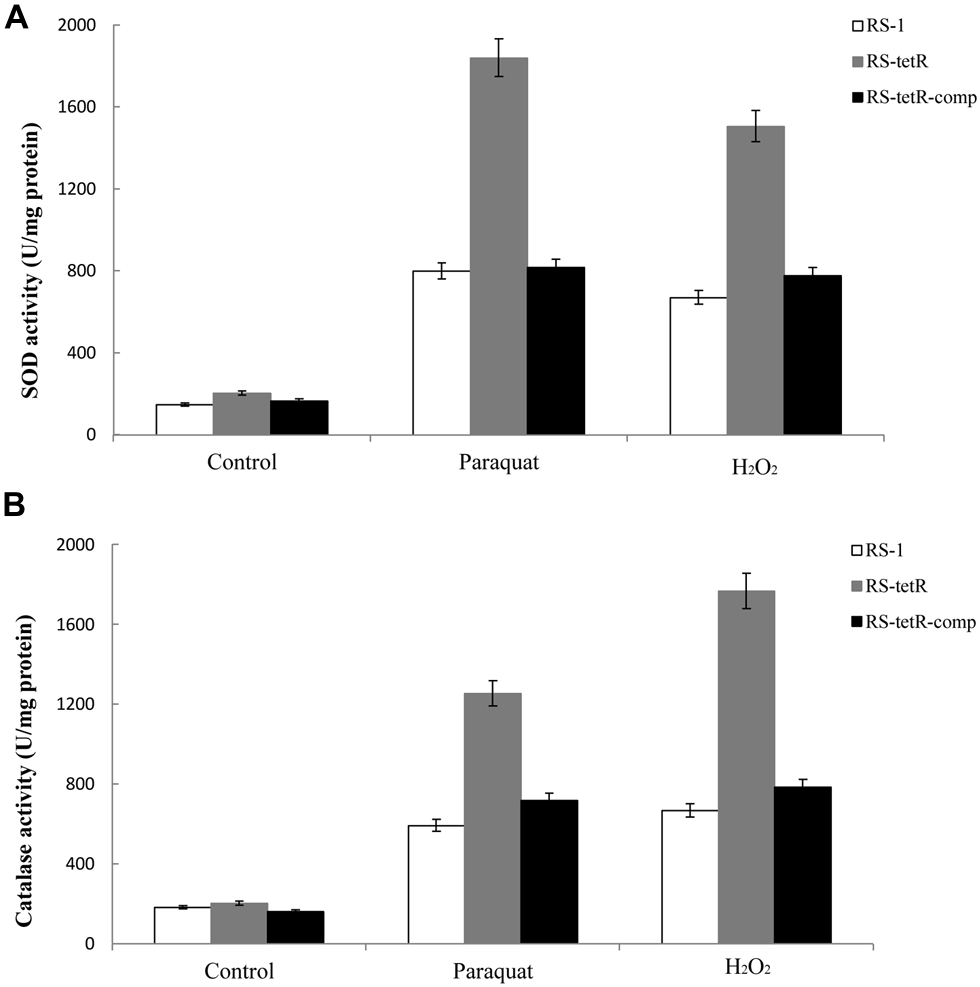
**Total SOD **(A)** and catalase **(B)** activities in response to superoxide and peroxide stress.** Enzyme activities of different strains were evaluated using cytoplasmic proteins after 60 min induction with oxidative compounds (0.4/0.8 mM paraquat or 40/60 mM H_2_O_2_, RS-1 and RS-*tetR*-comp/RS-*tetR*). Error bars indicated standard deviations from three independent experiments.

Superoxide dismutase and catalase in cytoplasm of bacterial cells play a pivotal role in protection against superoxide and peroxide stress (Henriques et al., 1998; Inaoka et al., 1999). They are highly efficient enzymes and likely to be transiently induced (Hassett and Cohen, 1989). Our results in **Figure 4** showed that their enzyme activities were increased in response to oxidative stress compared to the untreated control (**Figure 4**). More importantly, the RS-*tetR* mutant strain showed markedly higher levels of catalase and SOD activities compared to wild-type and complemented strains (**Figure 4**). The quantitative analysis of SOD activity showed that the absence of TetR led to ∼2.3-fold increase in enzyme activity in paraquat treatment and ∼2.2-fold in the H_2_O_2_ treatment. The quantitative catalase activity analysis yielded similar results: deletion of *tetR* resulted in ∼2.7-fold and ∼2.1-fold increase in H_2_O_2_-treated and paraquat-treated samples, respectively. These results raised the question as to whether TetR can also influence expression of genes encoding other oxidative stress detoxifying enzymes, such as *sodAB*, *katA*, *ahpCF* and *hemAXCDBL* (Zuber, 2009). We therefore carried out LC-MS and quantitative PCR analysis to address this issue.

### IDENTIFICATION OF PROTEINS REGULATED BY tetR

**FIGURE 5:**
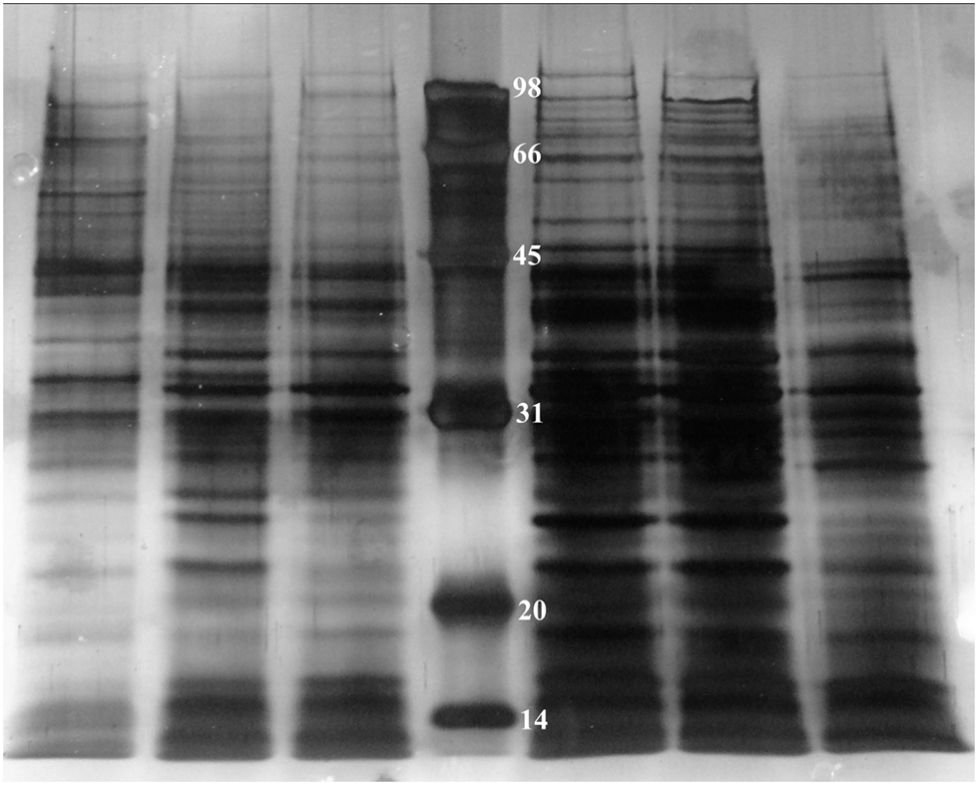
**1D SDS-PAGE of cytoplasmic proteins obtained from *A. avenae* subsp. *avenae* cells.** Lane 1–3, wild-type strain RS-1 under no stress conditions, 0.4 mM paraquat and 40 mM H_2_O_2_ stress conditions, respectively; Lane 4–6, mutant strain RS-*tetR* under 0.8 mM paraquat, 60 mM H_2_O_2_ stress conditions and no stress conditions, respectively.

In order to investigate how many proteins can be influenced by TetR under oxidative conditions, we employed 1D SDS-PAGE to compare cytoplasmic proteins of RS-1 and RS-*tetR*, prepared as described in Section “Materials and Methods” (**Figure 5**). The resultant protein bands were further digested and peptides were then analyzed by LC-MS/MS subsequently.

The protein profiling data of LC-MS/MS from two biological replicates revealed a non-redundant list of proteins, after removing overlapping entries, with high (>98%) confidence. The results clearly suggest differential expression of these newly identified cytoplasmic proteins between wild-type and *tetR* mutant strains. In total, there are 111 proteins identified (Tables S2 and S3). Specifically, 15 proteins were only identified in RS-1 while 96 proteins were identified only in RS-*tetR*. These proteins are mainly associated with oxidative stress response, detoxification, and biofilm-formation. COG enrichment suggested that there were more proteins involved in energy production and conversion, cell motility, post-translational modification in the RS-*tetR* strain, compared to RS-1 (*P* < 0.01, hypergeometric distribution test); whereas the wild-type strain produced more proteins involved in coenzyme transport and metabolism, amino acid transport and metabolism, and cell division (Figure S6). Overall, these results provide an explanation for the observed physiological changes caused by TetR and support our main hypothesis that this gene cluster plays a key role in bacterial survival under oxidative stress.

### QUANTITATIVE PCR ANALYSIS OF THE *tetR*-REGULATED GENES

**FIGURE 6:**
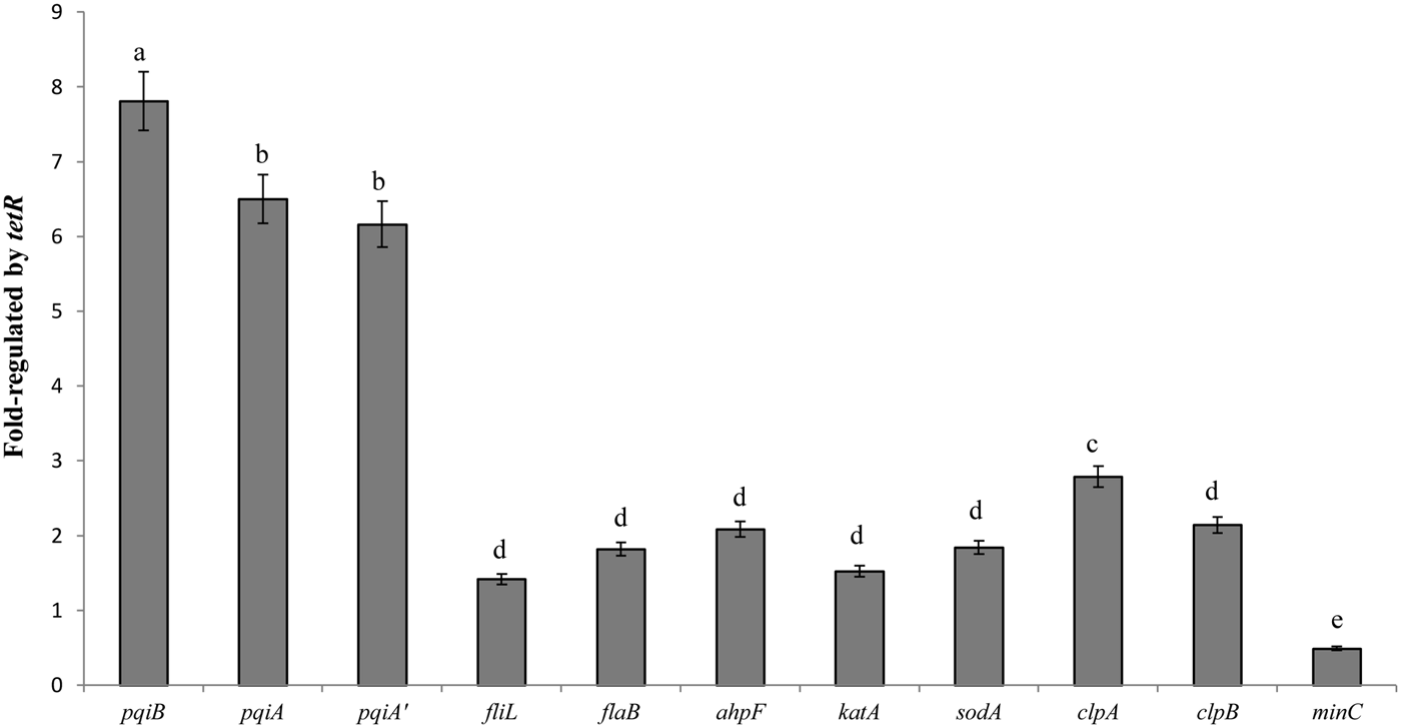
**Quantitative PCR analysis shown as fold change in gene expression regulated by *tetR*.** The data were quantified by the change-in-cycling-threshold method (2^-ΔΔCt^) using the 16S rRNA for normalization. Total RNA was extracted from RS-1 and RS-*tetR* cells exposed to the oxidative compounds for 10 min. Error bars indicated standard deviations from three independent experiments. Different letters indicated significant differences (*P* < 0.05) among treatments.

The LC-MS/MS study exhibited a comprehensive profile of proteins whose coding genes are regulated by the TetR regulator under oxidative stress. For further confirmation, we selected 11 genes from the proteomic profile which were involved in oxidative stress response or biofilm-formation, or encoded detoxifying enzymes. We examined their gene expression with quantitative real-time PCR. The results in **Figure 6** showed different fold change in gene expression regulated by *tetR* comparing mutant to wild-type strain. As expected, the expression of *pqiA’AB* was significantly up-regulated in the RS-*tetR* strain (6.2-, 6.5-, 7.8-fold, respectively). Similar results were obtained for genes *sodA*, *ahpF* and *katA*, which encode SOD, alkyl hydroperoxide reductase, and catalase, respectively (**Figure 6**). Up-regulated expression in these genes is expected to lead to increase in detoxifying enzyme activities, as shown in SOD and catalase activities assay (**Figure 6**). Moreover, the increased detoxifying enzyme activity can also contribute to the higher resistance to paraquat and H_2_O_2_. Flagella are important for bacterial colonization and biofilm formation (Conrad, 2012; Bogino et al., 2013; Ren et al., 2013). Quantitative real-time PCR showed that the gene expression level of *fliL* and *flaB*, two flagella-associated genes, was 1.4-fold and 1.8-fold higher in the absence of TetR. The *clpAB* gene family plays a role in synthesis of stable and native proteins, which are required for intracellular replication, stress tolerance, and biofilm formation of bacteria, in the presence of ATP (Wawrzynow et al., 2003; Frees et al., 2004). Thereby it was not surprising to see these genes up-regulated in the absence of the *tetR* regulator.

### TetR BINDS TO *pqiA’*-*tetR* INTERGENIC REGION

**FIGURE 7:**
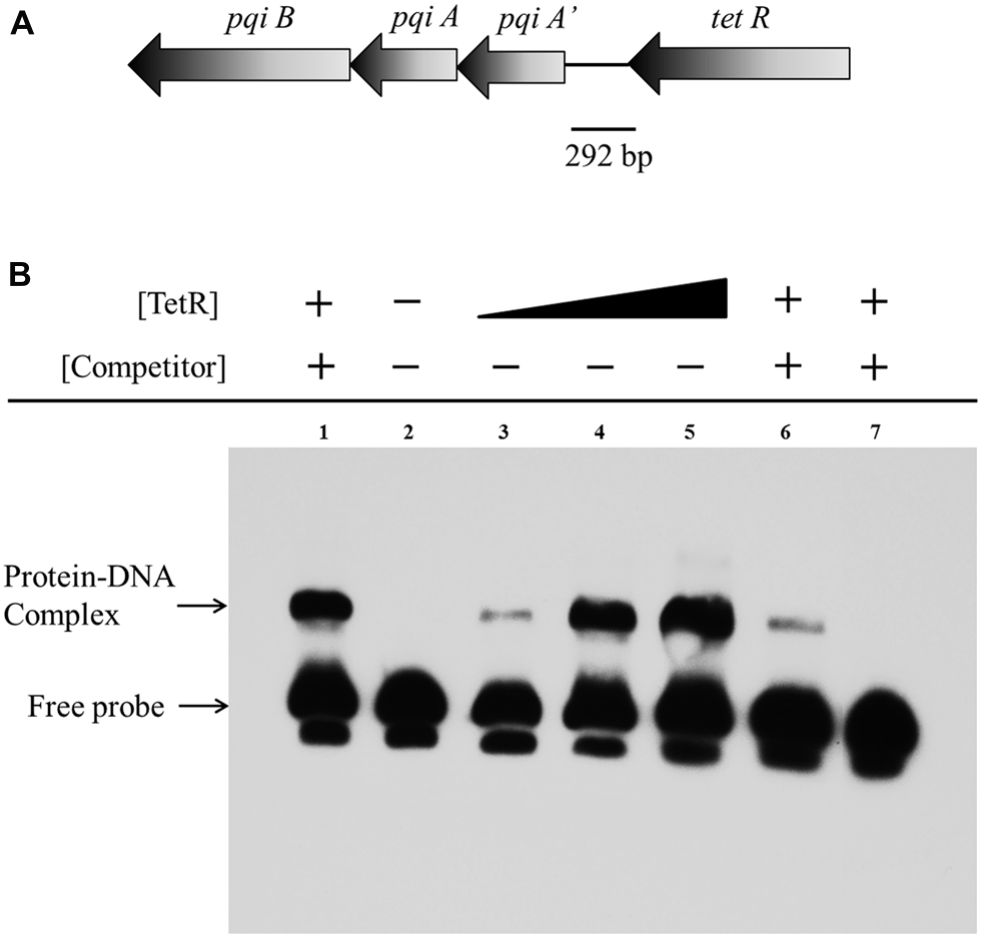
**Electrophoretic mobility shift assay of TetR binding to the *pqiA’-tetR* intergenic region. (A)** The 292 bp DNA fragment comprising the intergenic region between the *tetR* and *pqiA’* genes was amplified by PCR and used as probe in EMSAs. **(B)** Detection of protein–DNA complexes by EMSA using 0.05 pmol labeled probe incubated with increasing amount of GST-TetR protein (0 mg, Lane 2; 0.20 mg, Lane 3; 0.40 mg Lane 4; 0.60 mg, Lane 5). The 5 pmol unlabelled non-specific DNA, 0.05 and 5 pmol unlabeled specific DNA (cold competitor probe) was mixed and incubated with 0.40 mg purified GST-TetR protein before adding labeled specific probe in Lane 1, Lane 6 and Lane 7, respectively.

To investigate whether the repression of the genes on the gene cluster by TetR occurs via physical interaction between TetR and the promoter of the gene cluster, we performed EMSA using a 292 bp putative promoter fragment of the *pqiA’*-*tetR* intergenic region from *A. avenae* subsp. *avenae* RS-1 (**Figure 7A**). We purified the TetR protein with GST tag (about 48 kDa) and confirmed it by SDS-PAGE with silver staining (Figures S7A,B). After mixing the biotin-labeled probe with TetR, a band with substantial mobility shift corresponding to a TetR-DNA complex was detected (**Figure 7B**, Lane 3–5). When TetR concentration was increased, an increase in the shifted band was observed (**Figure 7B**, Lane 3–5). This result indicates that the binding of TetR to the DNA fragment is concentration dependent. As a control, we showed that there was no mobility shift of the probe when TetR was not added (**Figure 7B**, Lane 2). In addition, a strong binding shift appeared when unlabeled non-specific DNA was added (**Figure 7B**, Lane 1); while when unlabeled cold probe was added, it abolished the binding, demonstrating the binding sensitivity and specificity of TetR for this intergenic region (**Figure 7B**, Lane 6–7).

## DISCUSSION

It is believed that pathogenic bacteria can acquire and integrate foreign DNA into its chromosome. This process has been hypothesized to give advantages to the pathogenic bacteria (Dobrindt et al., 2004). *A. avenae* subsp. *avenae*, the phytopathogen causing BBS disease, has developed several mechanisms to defend itself against oxidative stress, including induction of protective enzymes against peroxide/superoxide and adjustment of eﬄux pumps. HGT is an efficient means to acquire foreign DNA, which helps to adjust cellular responses to successfully survive environmental challenges, such as oxidative stress.

Many methods have been developed to detect HGT including GC content, codon usage and phylogenetic tree analyses (Eisen, 2000). In these, phylogenetic tree has proven to be the “golden standard” for HGT detection (Bansal et al., 2012). Our phylogenetic tree analysis suggested that this gene cluster might be acquired from a *Pseudomonas* ancestor as many *Pseudomonas* species clustered with RS-1 (Figure S1). Consistent with this evidence, *A. avenae* RS-1 and most *Pseudomonas* strains are both soil dwellers; many *Pseudomonas* strains are plant pathogenic bacteria (Sarkar and Guttman, 2004). Thus, RS-1 may have gained these genes from a *Pseudomonas* donor.

In most cases, TetR-like transcriptional factors are believed to have two domains responsible for signal perception and DNA-binding, respectively (Hillerich and Westpheling, 2008). Besides, it was reported that the TetR protein regulates many genes and pathways in bacteria that are involved in secondary metabolism, MDR, and so on (Ramos et al., 2005; Hillerich and Westpheling, 2008). The deletion of the *tetR* gene in RS-1 rendered the bacterium more resistant to oxidative conditions, suggesting that it normally functions to regulate the onset of anti-oxidative stress response. Moreover, the LC-MS/MS results revealed that the TetR can regulate expression of multi-proteins in RS-1.

Biofilm-forming capacity of bacteria is important for their survival and infection on host cells. Therefore, studies on the regulatory mechanisms governing biofilm formation are important and could eventually lead to prevention of or therapy for pathogen infections. Nevertheless, the regulation of biofilm formation is too complex and multifactorial because of the varying environmental factors, including osmolarity, temperature, anaerobiosis, and levels of iron, ethanol, glucose, nitrites, and citrate (Prigent-Combaret et al., 1999; Schlag et al., 2007). In addition, there are a number of endogenous proteins, for instance CsrA, Crc, BfdR, and LuxR, which regulate biofilm-forming ability of bacteria (O’Toole et al., 2000; Jackson et al., 2002; Huang et al., 2013; HuiQiu et al., 2013). Here we reported that the *tetR* gene in a gene cluster of *A. avenae* subsp. *avenae* also affected its biofilm-forming ability. To access expression level of biofilm-forming associated genes, we employed LC-MS/MS analysis and real-time PCR study, and found activation of an array of flagellar genes and *clpAB* genes in the absence of TetR (Tables S2 and S3, **Figure 6**). On the other hand, the proteins down-regulated (such as methylcrotonoyl-CoA carboxylase and MinC) in the *tetR* mutant, mainly belong to bacterial catabolic pathways and regulator of cell division and growth (Tables S2 and S3; Höschle et al., 2005; Aguilar et al., 2008; Bramkamp and van Baarle, 2009; Lutkenhaus, 2009). In particular, the decreased *minC* gene expression can cause slower cell growth, which may help bacteria to withstand arduous conditions. These results also provide evidence supporting the role of TetR as a positive regulator of genes promoting cell growth.

As a transcriptional regulator, the TetR protein binds to the promoter of the gene cluster directly, as EMSA showed (**Figure 7**). Mutation of the *tetR* gene resulted in activation of the *pqiAA’B* genes, suggesting that TetR negatively controls the transcript levels of *pqiAA’B*. This result is consistent with our observations that the mutant displayed increased levels of resistance to paraquat and H_2_O_2_.

With the exception of TetR regulator, there are some other known regulators associated with oxidative stress regulation, such as OxyR or PerR. Although similar properties make them functionally analogous with each other, genetic evidence revealed that they have much difference on protein structures and functional mechanisms. OxyR, a member of the LysR family, is a well-characterized positive regulator of the adaptive response to H_2_O_2_ stress in *E. coli* and in *Salmonella enterica* serovar *typhimurium* (Zheng et al., 1998; Helmann, 2002), and a negative regulator of catalase expression in *Neisseria gonorrhoeae* (Tseng et al., 2003). Recently, more researches have shifted to favor a model for OxyR activation. It was believed a specific disulfide bond formation was required in the oxidative activation of OxyR, rather than being chemically modified by individual cysteine (Cys) residues (Helmann, 2002; Lee et al., 2004). PerR is a metal-dependent peroxide sensors that regulate inducible peroxide-defense genes, such as catalase gene *katA*, alkyl hydroperoxide reductase gene *ahpCF* (Horsburgh et al., 2001; Lee and Helmann, 2006). PerR is as well required for virulence and iron storage proteins in *Staphylococcus aureus* (Horsburgh et al., 2001). These features separate PerR functionally and mechanistically from TetR and OxyR regulators. Here, we did not find the TetR controlling virulence in *A. avenae* subsp. *avenae* by observing symptoms and analyzing lesion lengths 14 days post-inoculation (Figure S5). Most of the fully identified and characterized TetR-family repressors were associated with biosynthesis of antibiotics, eﬄux pumps, and osmotic stress (Aramaki et al., 1993, 1995). As a regulator of adaptive response to H_2_O_2_ stress, it was not known whether paraquat or H_2_O_2_ was the direct modulator for TetR protein activity. Future studies are required to cope with this question.

It is well-known that there is a balance between different factors contributing to fitness in bacteria. The trade-off hypothesis in pathogen evolution states that higher benefit in one aspect is correlated to lower benefit in another aspect (Alizon et al., 2009). Based on the trade-off hypothesis, there must be undesirable side effects derived from over-expression of the TetR-regulated genes. Our results showed that over-expression of the TetR-regulated genes in the *tetR* mutant led to low cell growth rate under non-oxidative stress conditions. In favorable growth conditions, fast growth would be more important to bacteria than keeping its defense heightened. TetR is a repressor in the operon to regulate the *pqiAA’B* genes to achieve the balance between fast growth and resistance to arduous surroundings. This feedback mechanism is of high flexibility, which is important to bacterial adaptability. TetR-like repressors, such as PrqR, Aur1R, EthR and CifR, with similar mechanisms are also found in other bacteria (Babykin et al., 2003; Engohang-Ndong et al., 2004; MacEachran et al., 2008; Novakova et al., 2010). In *Vibrio cholerae*, HapR (another TetR regulator) is found to play a positive role on detachment of *V. cholerae* to the gastrointestinal epithelium as well as a negative effect on biofilm formation (Silva et al., 2003). We still don’t know exactly the positive role of the *tetR* gene. However, based on the differential protein profiles between *tetR* mutant and wild-type strain, we hypothesize that TetR also plays positive roles, including promoting cell growth. More research is needed to reveal other physiological roles of TetR.

## Conflict of Interest Statement

The Review Editor Yuejin Hua declares that, despite being affiliated to the same institution as authors He Liu, Chun-Lan Yang, Meng-Yu Ge, Muhammad Ibrahim, Bin Li, Bo Zhu and Guan-Lin Xie, the review process was handled objectively and no conflict of interest exists. The authors declare that the research was conducted in the absence of any commercial or financial relationships that could be construed as a potential conflict of interest.
