# Supplementary material for: Regulatory role of tetR gene in a novel gene cluster of Acidovorax avenae subsp. avenae RS-1 under oxidative stress
Source: Front Microbiol. 2014 Oct 21;5:547. doi: 10.3389/fmicb.2014.00547 (PMC4204640; doi:10.3389/fmicb.2014.00547)
Supplement: Supplementary file 1 [file Data_Sheet_1.ZIP › Table.S3. 15 proteins identified in RS-1.pdf]

Table S3. 15 proteins identified in RS-1							
**Locus Tag**	**Protein name**	**Size (A.A)**	**Search score**	**Theoritical Mass**	**GRAVY**	**PI**	**COG**
Acav_0072	methylcrotonoyl-CoA carboxylase	663	92	70775.5	-0.086	6.35	0439
Acav_0157	septum site-determining protein MinC	257	62	26875.6	0.117	4.90	0850
Acav_0339	glutamate carboxypeptidase	430	60	45896.2	-0.136	6.81	0624
Acav_0349	glutamyl-tRNA(gln) amidotransferase subunit B	486	63	53159.3	-0.270	5.30	0064
Acav_0645	fumarylacetoacetate (FAA) hydrolase	336	28.1	36471.3	-0.197	5.08	0179
Acav_0888	family 5 extracellular solute-binding protein	526	135	58194.3	-0.304	9.11	0747
Acav_1326	iron-sulfur cluster-binding protein	365	46.2	39941.6	-0.235	7.68	1600
Acav_1488	putative transmembrane protein	196	27.3	21437.2	-0.357	6.93	NO
Acav_1589	3-oxoacyl-ACP reductase	267	41.6	27556	0.035	5.37	1028
Acav_1971	3-phosphoshikimate 1-carboxyvinyltransferase	679	136	72729.4	0.053	6.14	0128
Acav_2479	vitamin-B12 independent methionine synthase	341	83.2	38009.6	-0.174	5.89	0620
Acav_2601	butyryl-CoA dehydrogenase	388	59	41392.8	-0.094	5.47	1960
Acav_2701	acetyl-CoA carboxylase, biotin carboxylase	2049	468	165856.9	0.899	4.84	0439
Acav_2911	aspartate transaminase	394	27.7	43047	-0.003	5.9	0436
Acav_4004	acyl-CoA dehydrogenase domain-containing pro	601	23.5	65523	-0.157	6.01	1960
