# Supplementary material for: Regulatory role of tetR gene in a novel gene cluster of Acidovorax avenae subsp. avenae RS-1 under oxidative stress
Source: Front Microbiol. 2014 Oct 21;5:547. doi: 10.3389/fmicb.2014.00547 (PMC4204640; doi:10.3389/fmicb.2014.00547)
Supplement: Supplementary file 1 [file Data_Sheet_1.ZIP › supplementary figures.pdf]

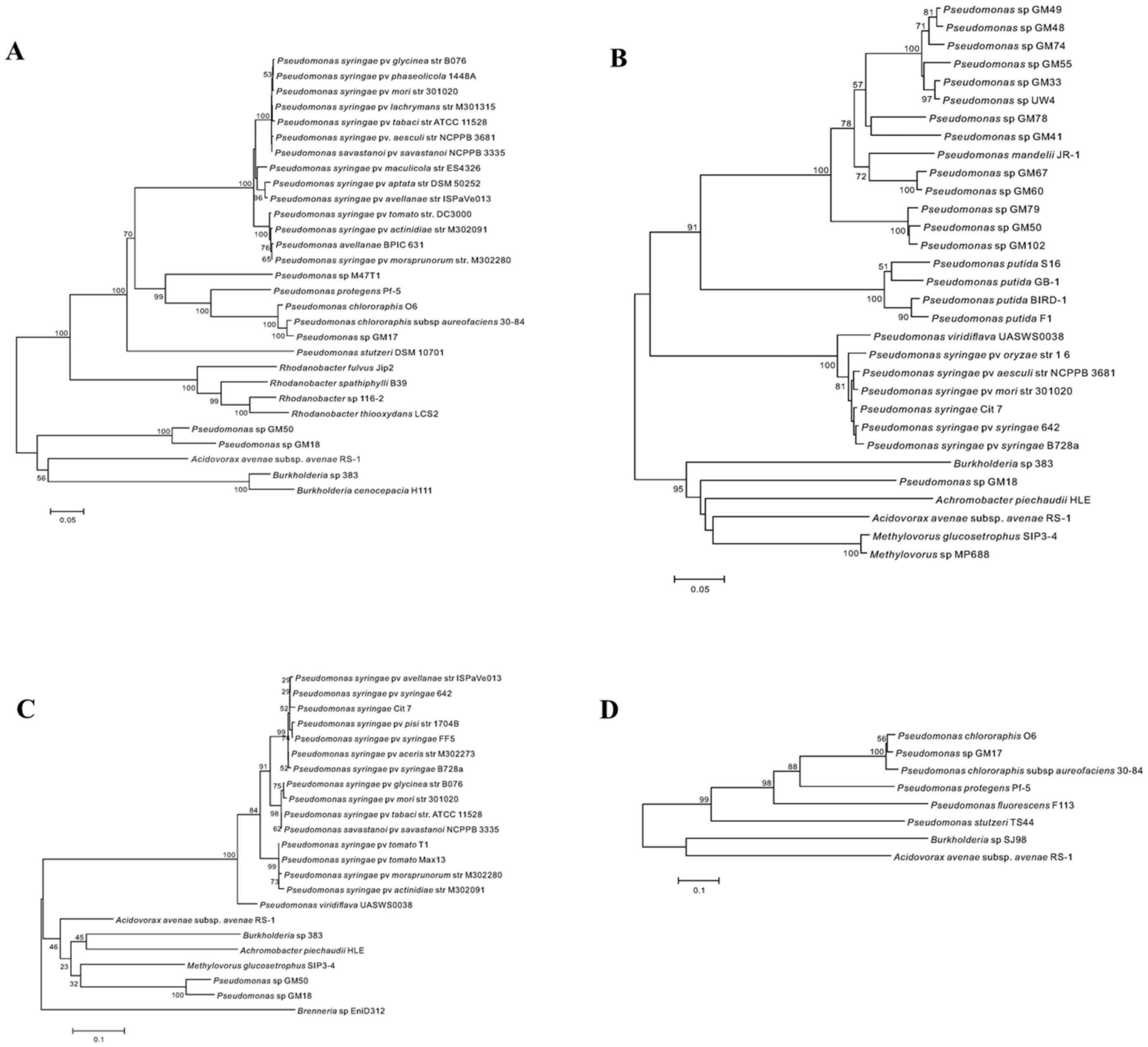

Figure S1. **Phylogenetic analysis of genomic island in *A. avenae* subsp. *Avenae* strain RS-1**. Maximum Likelihood (ML) phylogenies were constructed by Phyml using a JTT model and a gamma distribution with eight rate categories. 1,000 bootstraps were performed to gain the branch support values. a, RS-1088 (*pqiB*); b, RS-1089 (*pqiA*); c, RS-1090 (*pqiA'*); d, RS-1091 (*tetR*).

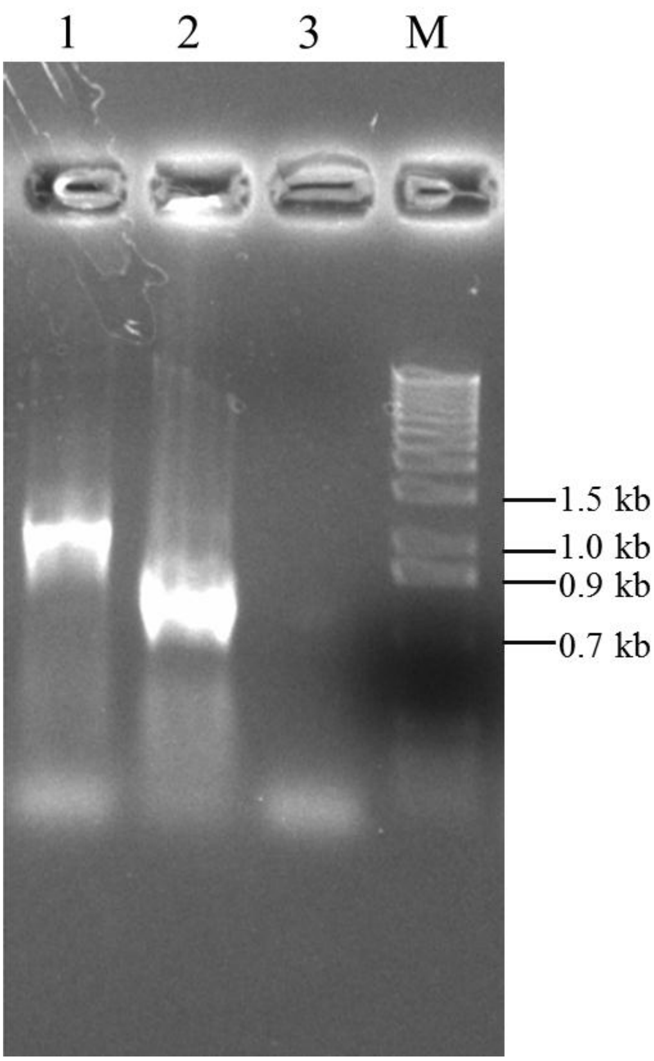

Figure S2. Identification of *tetR*-deletion mutant by PCR amplification. Double exchange homologous recombination event was occurred using suicide plasmid pKMS1. 1062 bp fragment was amplified with tetR-comp-F/R primer pair and wild-type strain RS-1 genomic DNA as template (Lane 1). 795 bp fragment was amplified with the same primer pair and *tetR*-deletion strain RS-*tetR* genomic DNA as template (Lane 2). pKMS1 vector was used as negative control (Lane 3). PCR product size was shown by DNA marker (Lane 4).

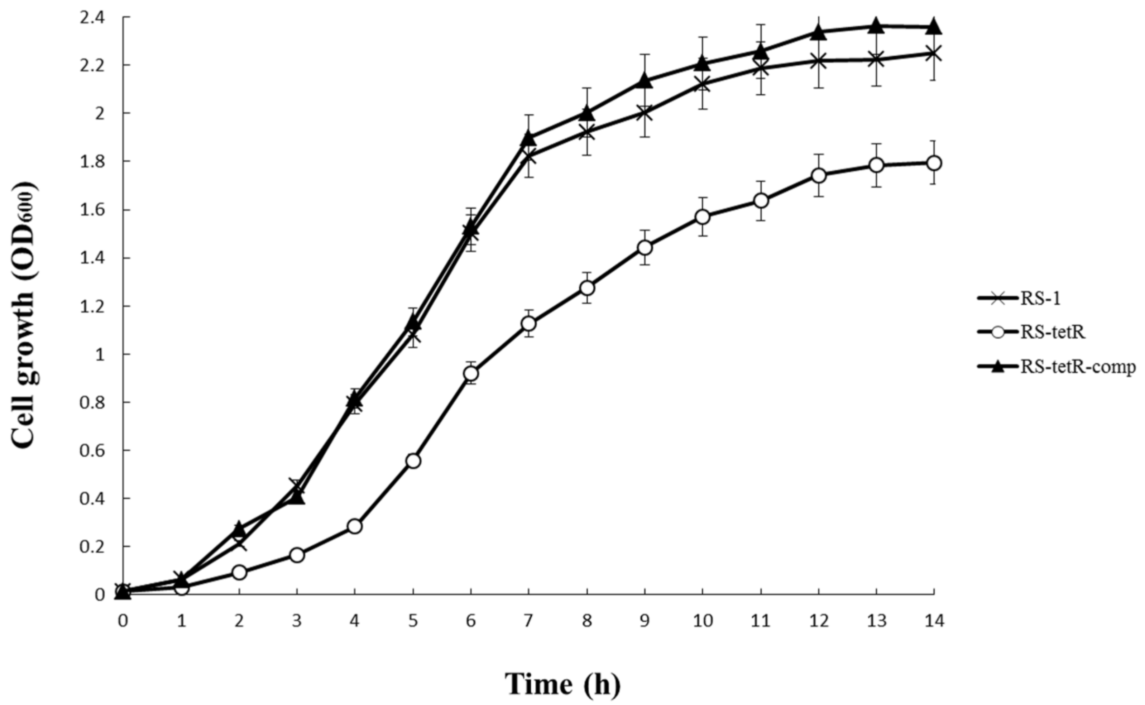

Figure S3. **Comparison of growth kinetics**. To monitor growth, overnight cultures were diluted into LB broth to an optical density (600 nm) of 0.05 and then measured every one hour. The mutant strain RS-*tetR* grew slowly than the wild-type and complementary strains. This experiment was reproduced three times.

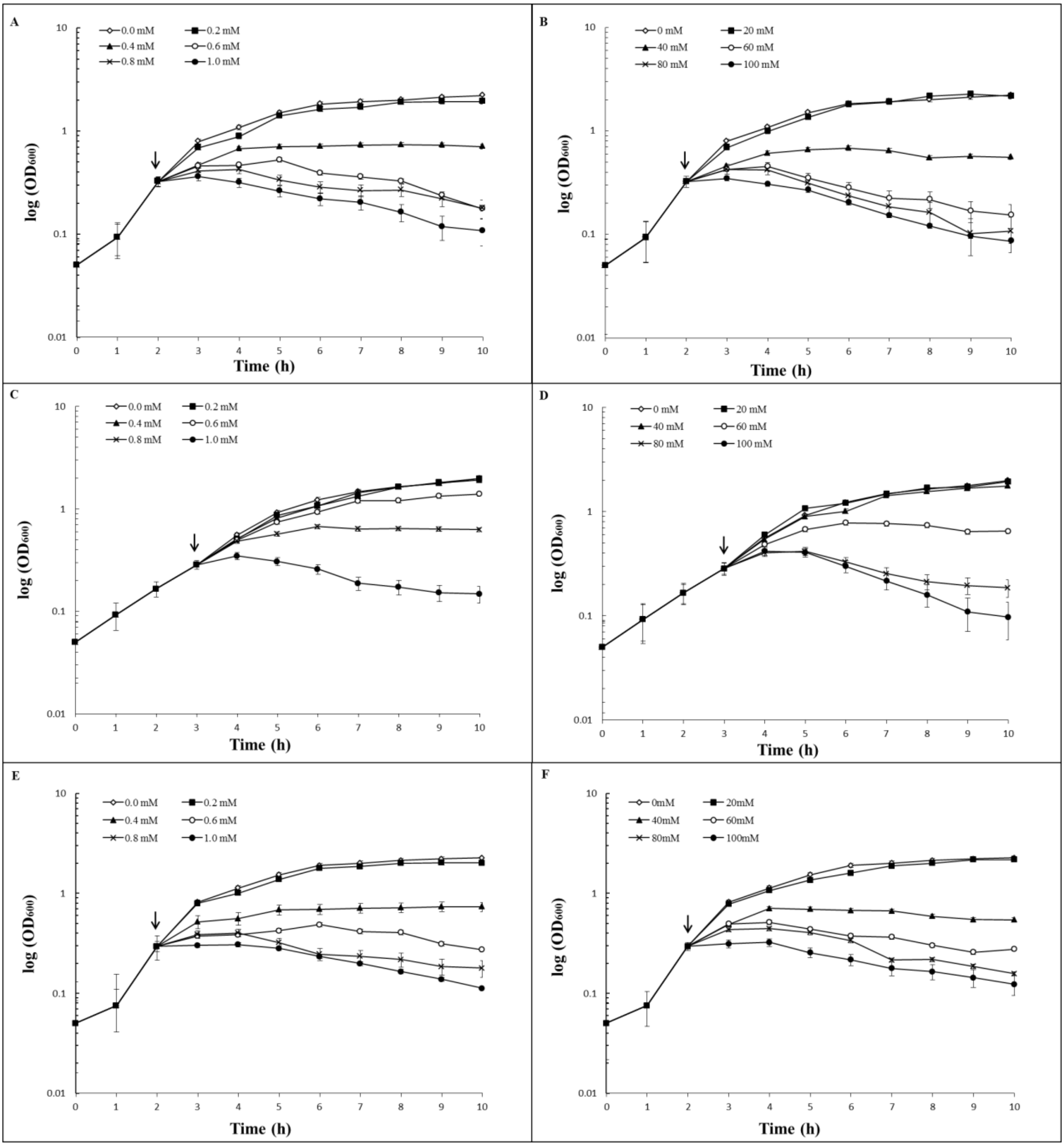

Figure S4. Growth kinetics of wild-type strain RS-1 (A & B), mutant strain RS-*tetR* (C & D), and complemented strain RS-*tetR*-comp (E & F) in response to oxidative stress. The reactive oxygen reagent (H_2O_2 or paraquat) was added at mid-log phase (OD600=0.3) with the indicated final concentrations. The growth curves reflected different sensitivities of the cells to the compounds by comparison with an untreated control. Arrows showed the time point at which the compound was added. **(A) (C)** and **(E)**, RS-1, RS-*tetR*, and RS-*tetR*-comp, respectively, treated by 0.0 mM (△), 0.20 mM (■), 0.4 mM (▲), 0.6 mM (○), 0.8 mM (×), and 1.0 mM (●) paraquat; **(B) (D)** and **(F)**, RS-1, RS-*tetR*, and RS-*tetR*-comp, respectively, treated by 0 mM (△), 20 mM (■), 40 mM (▲), 60 mM (○), 80 mM (×), and 100 mM (●) H_2O_2. Error bars indicated standard deviations from the mean of triple independent experiments.

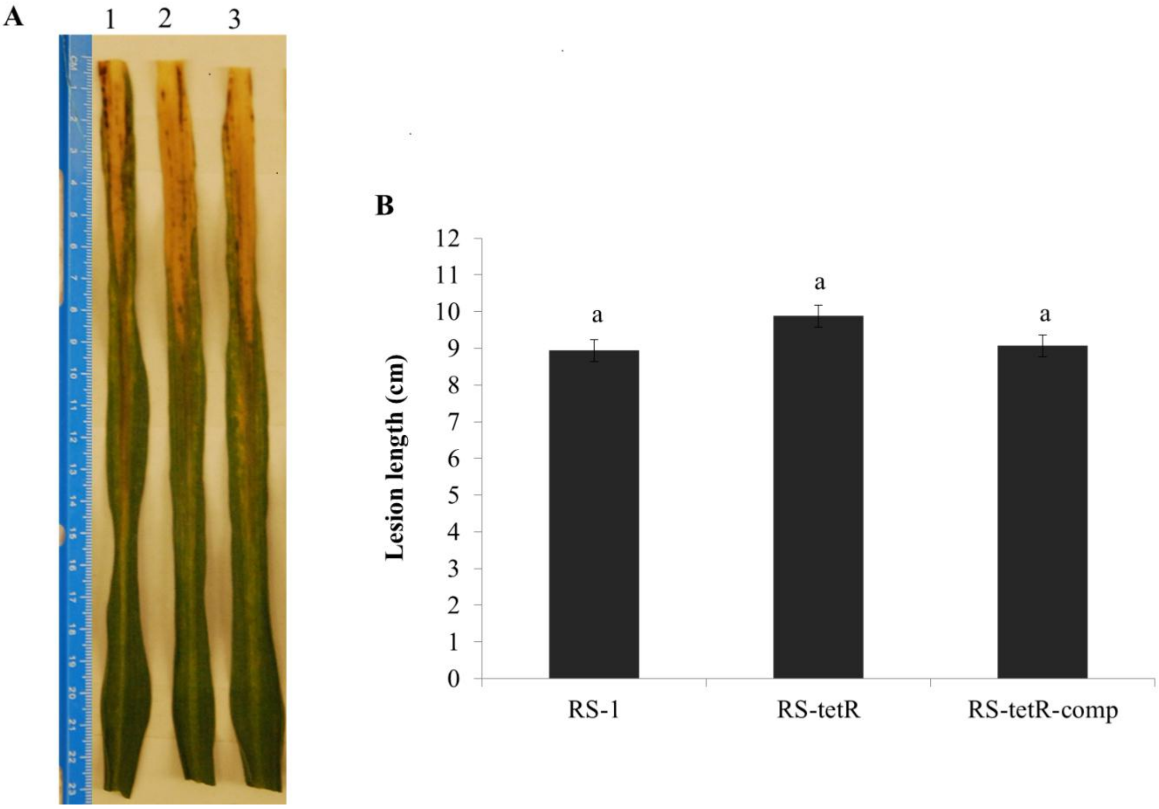

Figure S5. Symptoms of 14 days post inoculation and measurement of lesion lengths. a, 6 weeks-old rice leaves were inoculated by wild-type strain RS-1 (lane 1), mutant strain RS-*tetR* (lane 2), and complementary strain RS-*tetR*-comp (lane 3) and were photographed 14 days after leaf-clip inoculation. b, Bacterial virulence was scored by measuring and analysis of lesion lengths. Means with same letter showed results without significant difference (P<0.05). Three independent repeats showed similar results.

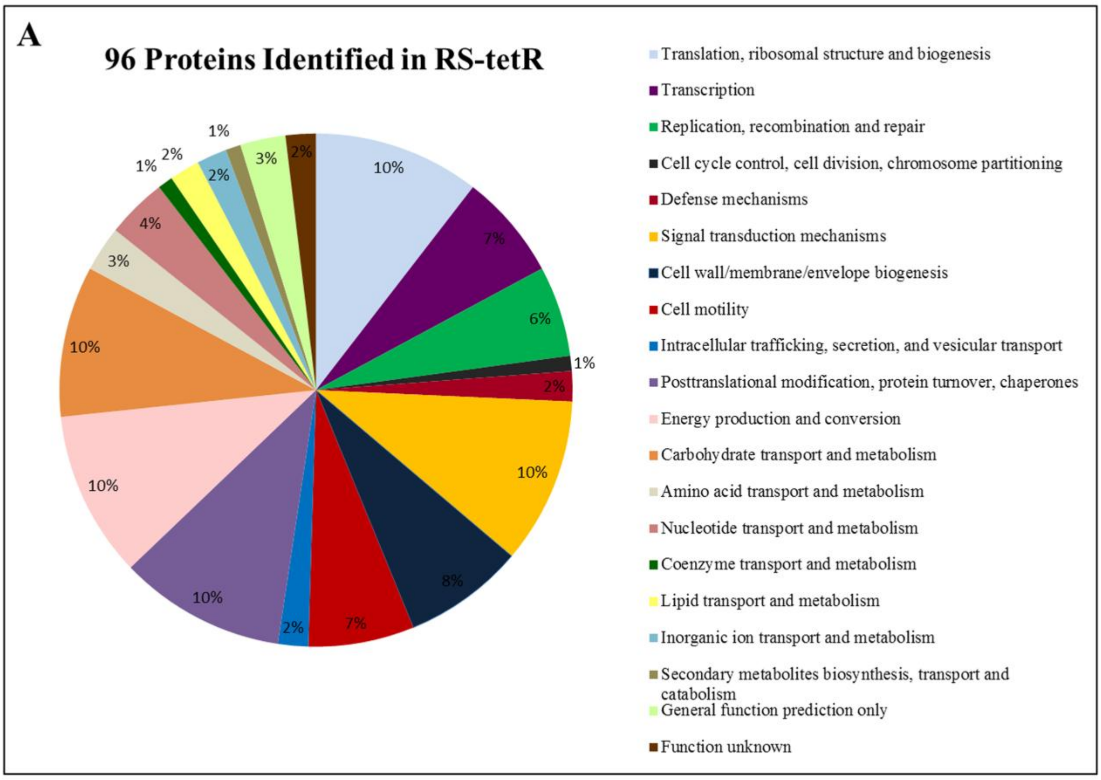

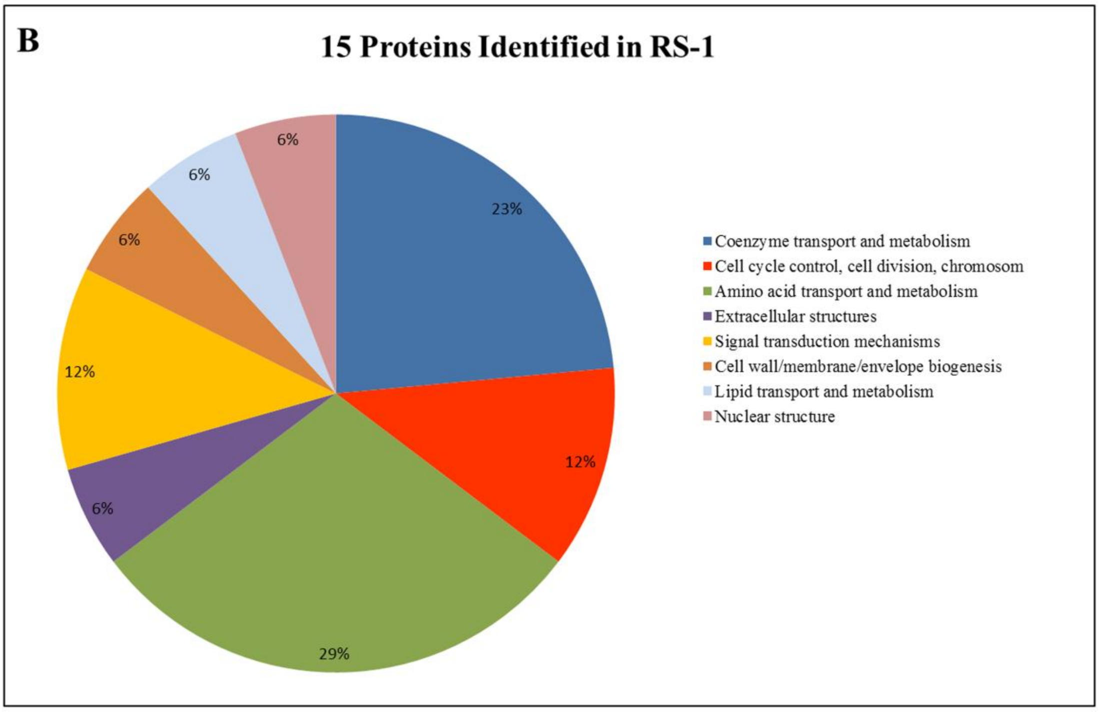

Figure S6. Pie graph showing the different protein clusters according to LC-MS/MS results. a, the proteins identified in RS-*tetR* were grouped into 19 clusters; b, the proteins identified in RS-1 were grouped into 8 clusters.

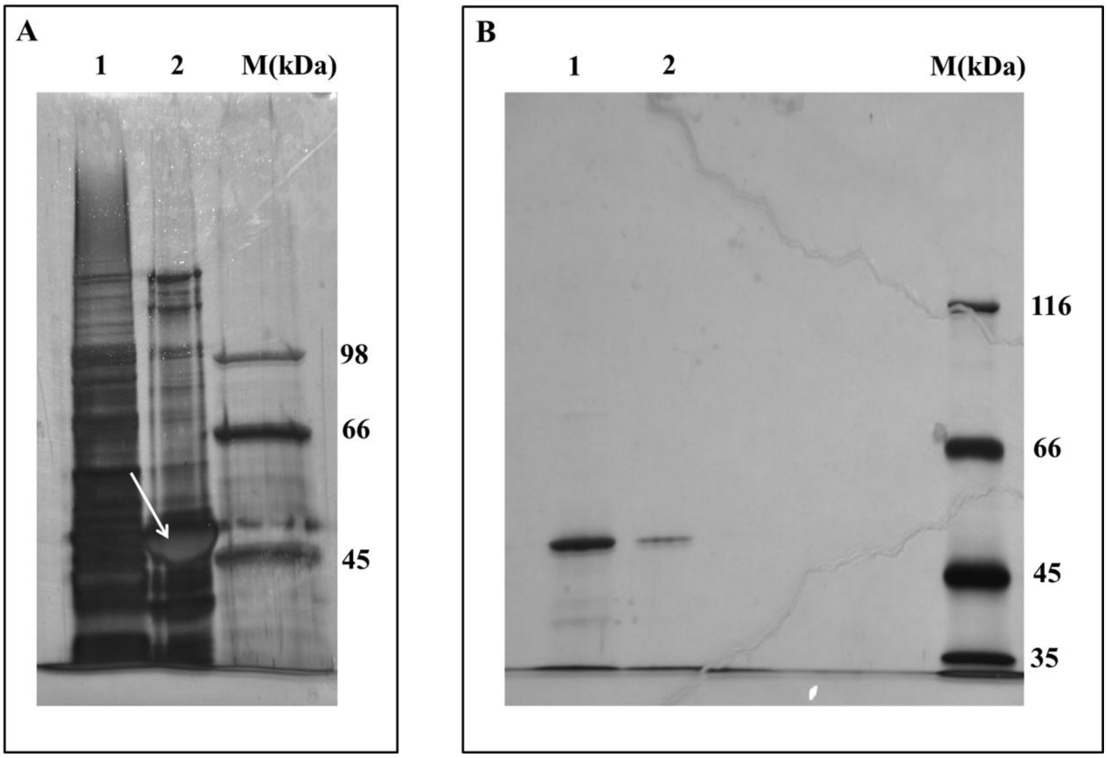

Figure S7. The overexpressed and purified TetR protein with GST-tag. a, Protein expressed in *E.coli* BL21(DE3). Lane 1, protein from cellular debris; Lane 2, protein from bacterial supernatant. Arrow indicates the TetR protein overexpressed in *E.coli* BL21(DE3). b, Lane1 and 2, The TetR protein with GST-tag was separated and purified by GST-tag Bind resin.
