# Supplementary material for: Regulatory role of tetR gene in a novel gene cluster of Acidovorax avenae subsp. avenae RS-1 under oxidative stress
Source: Front Microbiol. 2014 Oct 21;5:547. doi: 10.3389/fmicb.2014.00547 (PMC4204640; doi:10.3389/fmicb.2014.00547)
Supplement: Supplementary file 1 [file Data_Sheet_1.ZIP › Table.S1.pdf]

Table S1. List of oligo nucleotide polymerase chain reaction (PCR) primers used in this study

Primer name	Nucleotide sequence (5'-3') [a]	Target PCR product of function
tetR1-F	CACACAGGATCCTTGTTTCGGGCGCCTCTG (B)	250 bp internal upstream fragment of *tetR*; used to create mutant RS-tetR
tetR1-R	CCGGAATTCGCGGTGGACATTGACTGC (E)	
tetR2-F	CCGGAATTCTTGAGGACTATGTTGCGGTGAT(E)	186 bp internal downstream fragment of *tetR*; used to create mutant RS-tetR
tetR2-R	CATAGAAGCTTACGGCCTGTGGGCTCTGT(H)	
tetR-comp-F	TGACATATGTTGTTTCGGGCGCCTCTGCTGG (N)	1062 bp fragment using for complementation of *tetR* mutant
tetR-comp-R	TGAGGATCCATGGCCTTTCTTGCCTGATCCG (B)	
tetR-F	ATATAGGATCCTTGTTTCGGGCGCCTCTGCT (B)	763 bp *tetR*-containing DNA fragment using for expressing TetR protein
tetR -R	TGTCAGTCGACTCAATGGCCTTTCTTGCCTG (S)	
GI-prom-F	CAGGAAGGGCACGATGAT	292 bp fragment of the *pqiA'-tetR* intergenic region using for EMSA
GI-prom-R	GGTCGAGGCTCTGGTCAT	
Unspecific-F	AAGCCCATGCTCCAATCCG	150 bp fragment using as unspecific DNA control in EMSA
Unspecific-R	ACGACTCGCCGTGCTCCTG	
16S rRNA-R	TAGCTCAGGTGGTTAGAGCGC	Reference gene used in real-time PCR
16S rRNA-F	CAACGCGAACATACGACTCAA	
pqiB-F	GTGTCTGCCCGGATCGTAGT	273 bp fragment of *pqiB* gene using for real-time PCR
pqiB-R	AAGACCCAGTCGGTTTCCAC	
pqiA-F	CACCACGCCGAAGAAGAAAA	199 bp fragment of *pqiA* gene using for real-time PCR
pqiA-R	GGCCCTGCTGCTGTGGACTT	
PqiA'-F	GGTGGACAACTCCCACAGCC	287 bp fragment of *pqiA'* gene using for real-time PCR
PqiA'-R	TGTCCGTGATCATCGTGCCCTTCCT	
fliL-F	TCCTCATCATCGCCATCGTC	289 bp fragment of *fliL* gene using for real-time PCR
fliL-R	ATGCTGGGCAGGTACTGTTT	
flaB-F	GTCCGACAAGGGCTACACG	201 bp fragment of *flaB* gene using for real-time PCR
flaB-R	TCGAACCGGGACTGGAGTG	
ahpF-F	CCGCAAGCCGTCCTTCACT	228 bp fragment of *ahpF* gene using for real-time PCR
ahpF-R	ACCACGTCCGGGCAGTTGT	
katA-F	AACCGCTGGCCCGACAACT	199 bp fragment of *katA* gene using for real-time PCR
katA-R	TGTGGAACGGGCACTTGG	
sodA-F	CCCTATGCCTATGACGCC	241 bp fragment of *sodA* gene using for real-time PCR
sodA-R	CCATCACGGTCCAGAACA	
clpA-F	GACCATCGAGCGTGACCT	234 bp fragment of *clpA* gene using for real-time PCR
clpA-R	TGGCGCTCCATGTACTCC	
clpB-F	TGTCCCACCGCTACATCA	185 bp fragment of *clpB* gene using for real-time PCR
clpB-R	CGAGGCTTCGTCCTTTTC	
minC-F	CGTCGTGGTGGACAAACC	197 bp fragment of *minC* gene using for real-time PCR
minC-R	CCAGGCAGGTGCTGAAGAT	

[a] Underlined nucleotides in some of the PCR primers represent restriction sites of enzymes indicated in parentheses (E = *Eco*RI; B = *Bam*HI; H = *Hind*III; N = *Nde*I; S = *Sal*I). The corresponding enzymes were used to excise the obtained PCR products

from the cloning vector pGEM-T and clone them into plasmid pKMS1, pRADK or pGEX6P-1 for in-frame deletion mutagenesis, complementation or protein expression, respectively.
