# Supplementary material for: Regulatory role of tetR gene in a novel gene cluster of Acidovorax avenae subsp. avenae RS-1 under oxidative stress
Source: Front Microbiol. 2014 Oct 21;5:547. doi: 10.3389/fmicb.2014.00547 (PMC4204640; doi:10.3389/fmicb.2014.00547)
Supplement: Supplementary file 1 [file Data_Sheet_1.ZIP › Table.S2. 96 proteins identified in RS-tetR.pdf]

| TABLE S2. 96 proteins identified in RS-tetR | | | | | | | |
|---|---|---|---|---|---|---|---|
| **Locus Tag** | **Protein name** | **Size (A.A)** | **Search score** | **Theoritical Mass** | **GRAVY** | **PI** | **COG** |
| Acav_0003 | DNA gyrase subunit B | 877 | 79 | 95912.6 | -0.362 | 5.70 | 0187 |
| Acav_0010 | HsdR family type I site-specific deoxyribonuclease | 1098 | 345 | 123196.7 | -0.269 | 5.96 | 0610 |
| Acav_0357 | penicillin-binding protein 2 | 648 | 114 | 71079.6 | -0.282 | 8.61 | 0768 |
| Acav_0403 | translation elongation factor G | 700 | 935 | 77411.5 | -0.289 | 5.22 | 0480 |
| Acav_0580 | family 1 extracellular solute-binding protein | 577 | 85 | 64233.1 | -0.498 | 8.08 | 1653 |
| Acav_0742 | heat shock protein HslVU, ATPase subunit HslU | 438 | 65 | 48687.5 | -0.342 | 5.51 | 1220 |
| Acav_0747 | UDP-N-acetylmuramyl tripeptide synthetase | 519 | 181 | 54496.6 | -0.017 | 5.64 | 0769 |
| Acav_0753 | UDP-N-acetylmuramate--L-alanine ligase | 475 | 87 | 50469.8 | 0.158 | 5.89 | 0773 |
| Acav_0807 | MiaB family RNA modification protein | 449 | 73 | 49696.8 | -0.183 | 6.00 | 0621 |
| Acav_0866 | binding-protein-dependent transport system inner membran | 543 | 60 | 58150.0 | 0.760 | 10.76 | 1178 |
| Acav_1108 | urate catabolism protein | 322 | 62 | 36586.2 | -0.522 | 5.91 | 0726 |
| Acav_1161 | aconitate hydratase 1 | 963 | 81 | 104778.7 | -0.162 | 6.67 | 1048 |
| Acav_1219 | lysyl-tRNA synthetase | 518 | 74 | 58205.0 | -0.404 | 5.47 | 1190 |
| Acav_1243 | chaperone protein DnaK | 654 | 203 | 69700.0 | -0.310 | 5.05 | 0443 |
| Acav_1256 | Homoserine dehydrogenase | 444 | 59 | 46855.0 | 0.246 | 5.53 | 0460 |
| Acav_1267 | ATP-dependent chaperone ClpB | 869 | 126 | 95709.5 | -0.315 | 5.54 | 0542 |
| Acav_1278 | polyribonucleotide nucleotidyltransferase | 759 | 97 | 82293.9 | -0.238 | 5.57 | 1185 |
| Acav_1288 | NADH-quinone oxidoreductase subunit G | 720 | 92 | 76130.0 | 0.037 | 6.18 | 1034 |
| Acav_1300 | ABC transporter | 236 | 124 | 25744.8 | 0.053 | 6.32 | 0410 |
| Acav_1341 | enolase | 428 | 64 | 45800.8 | -0.091 | 4.82 | 0148 |
| Acav_1409 | peroxiredoxin | 191 | 71 | 21002.9 | -0.209 | 5.87 | 0450 |
| Acav_1410 | alkyl hydroperoxide reductase subunit F | 522 | 110 | 56082.7 | -0.060 | 5.37 | 3634 |
| Acav_1417 | phosphoenolpyruvate synthase | 796 | 1077 | 86319.2 | -0.117 | 5.16 | 0574 |
| Acav_1496 | anti-sigma H sporulation factor LonB | 808 | 217 | 89371.1 | -0.304 | 6.32 | 0466 |
| Acav_1507 | FHA domain-containing protein | 785 | 62 | 79330.9 | 0.028 | 4.81 | 3456 |
| Acav_1530 | class II fumarate hydratase | 461 | 198 | 49210.8 | -0.149 | 6.23 | 0114 |
| Acav_1539 | acetate/CoA ligase | 664 | 432 | 72083.3 | -0.113 | 6.04 | 0365 |
| Acav_1627 | transcription-repair coupling factor | 1163 | 188 | 129682.0 | -0.269 | 5.95 | 1197 |

| | | | | | | | |
|---|---|---|---|---|---|---|---|
| Acav_1631 | winged helix family two component transcriptional regulato | 243 | 278 | 27174.3 | -0.225 | 5.87 | 0745 |
| Acav_1653 | 2-dehydro-3-deoxygluconokinase | 321 | 66 | 33865.4 | -0.009 | 5.73 | 0524 |
| Acav_1696 | histone family protein DNA-binding protein | 90 | 111 | 9511.1 | -0.316 | 10.67 | 0776 |
| Acav_1721 | aldehyde dehydrogenase | 497 | 112 | 52342.8 | 0.074 | 6.10 | 1012 |
| Acav_1744 | peptidase S1 and S6 chymotrypsin/Hap | 788 | 112 | 86151.7 | -0.330 | 6.47 | 0265 |
| Acav_1792 | peptidase A2A | 233 | 61 | 23935.3 | 0.082 | 9.18 | 3577 |
| Acav_1810 | Tex-like protein | 788 | 541 | 86416.6 | -0.286 | 6.43 | 2183 |
| Acav_1854 | translation initiation factor IF-2 | 946 | 622 | 101510.8 | -0.434 | 6.06 | 0532 |
| Acav_1857 | GTP-binding protein TypA | 608 | 91 | 66752.2 | -0.264 | 5.33 | 1217 |
| Acav_1868 | inosine-5'-monophosphate dehydrogenase | 489 | 110 | 51635.3 | 0.044 | 7.10 | 0516 |
| Acav_1871 | GMP synthase large subuni | 541 | 127 | 59291.1 | -0.057 | 6.07 | 0519 |
| Acav_1905 | ImpA family type VI secretion-associated protein | 848 | 103 | 92844.7 | -0.484 | 5.78 | 3501 |
| Acav_1933 | delta-1-pyrroline-5-carboxylate dehydrogenase | 1247 | 67 | 131598.6 | 0.051 | 6.25 | 1012 |
| Acav_2002 | dihydrolipoamide dehydrogenase | 475 | 73 | 50057.3 | 0.015 | 6.05 | 1249 |
| Acav_2010 | ATP-dependent Clp protease ATP-binding protein ClpA | 782 | 61 | 85643.0 | -0.254 | 6.03 | 0542 |
| Acav_2020 | aldehyde dehydrogenase | 506 | 127 | 55165.1 | -0.089 | 5.93 | 1012 |
| Acav_2153 | catalase/peroxidase HPI | 743 | 82 | 81132.9 | -0.418 | 5.58 | 0376 |
| Acav_2182 | UTP-GlnB uridylyltransferase, GlnD | 868 | 60 | 97528.3 | -0.243 | 7.78 | 2844 |
| Acav_2385 | phenylalanyl-tRNA synthetase subunit beta | 812 | 119 | 88671.5 | -0.098 | 5.63 | 0072 |
| Acav_2396 | chromosome segregation protein SMC | 1175 | 354 | 130606.1 | -0.541 | 5.13 | 1196 |
| Acav_2398 | NAD-dependent DNA ligase | 732 | 547 | 78805.7 | -0.172 | 6.04 | 0272 |
| Acav_2423 | phosphoglucomutase | 462 | 63 | 49836.6 | -0.129 | 5.44 | 1109 |
| Acav_2425 | three-deoxy-D-manno-octulosonic-acid transferase domain | 422 | 60 | 50311.0 | -0.012 | 8.49 | 1519 |
| Acav_2592 | carbamoyl-phosphate synthase large subunit | 1086 | 223 | 118444.7 | -0.147 | 5.16 | 0458 |
| Acav_2599 | methylmalonate-semialdehyde dehydrogenase | 507 | 75 | 53583.6 | 0.096 | 5.96 | 1012 |
| Acav_2628 | sulfatase | 566 | 60 | 62498.1 | 0.096 | 8.68 | 2194 |
| Acav_2644 | tetraacyldisaccharide 4'-kinase | 346 | 63 | 37142.8 | -0.007 | 10.00 | 1663 |
| Acav_2752 | dihydrolipoamide dehydrogenase | 618 | 125 | 63563.1 | 0.116 | 5.90 | 1249 |
| Acav_2753 | pyruvate dehydrogenase complex dihydrolipoamide acetylt | 565 | 74 | 58487.1 | -0.017 | 5.67 | 0508 |
| Acav_2760 | ribose 5-phosphate isomerase | 230 | 68 | 24274.8 | 0.100 | 6.09 | 0120 |
| Acav_2810 | cysteinyl-tRNA synthetase | 459 | 128 | 50814.8 | -0.244 | 5.90 | 0215 |

| | | | | | | | |
|---|---|---|---|---|---|---|---|
| Acav_2951 | methyl-accepting chemotaxis sensory transducer with Cach | 536 | 59 | 57317.9 | -0.095 | 6.00 | 0840 |
| Acav_3096 | multi-sensor hybrid histidine kinase | 1624 | 278 | 176676.8 | -0.114 | 5.82 | 0642 |
| Acav_3137 | trehalose synthase | 1142 | 95 | 128666.5 | -0.350 | 5.19 | 0366 |
| Acav_3161 | YccS/YhfK family integral membrane protein | 750 | 209 | 80712.2 | 0.277 | 9.35 | 1289 |
| Acav_3167 | ABC transporter | 576 | 67 | 60984.0 | -0.190 | 6.35 | 0488 |
| Acav_3170 | valyl-tRNA synthetase | 961 | 757 | 106771.3 | -0.269 | 5.81 | 0525 |
| Acav_3186 | ferredoxin--NAD(+) reductase | 356 | 85 | 38560.8 | -0.201 | 6.73 | 0543 |
| Acav_3459 | CheR-type MCP methyltransferase | 267 | 65 | 29913.2 | -0.228 | 9.61 | 1352 |
| Acav_3496 | (p)ppGpp synthetase I SpoT/RelA | 755 | 227 | 83534.5 | -0.239 | 8.49 | 0317 |
| Acav_3524 | 4-diphosphocytidyl-2-C-methyl-D-erythritol kinase | 285 | 69 | 30818.1 | -0.056 | 5.92 | 1947 |
| Acav_3551 | response regulator receiver | 580 | 62 | 63479.4 | -0.091 | 6.03 | 0784 |
| Acav_3555 | aspartyl-tRNA synthetase | 604 | 69 | 67885.5 | -0.330 | 5.82 | 0173 |
| Acav_3604 | preprotein translocase subunit SecA | 922 | 648 | 103398.4 | -0.491 | 5.25 | 0653 |
| Acav_3614 | lipoprotein releasing system, ATP-binding protein | 230 | 72 | 24544.1 | 0.066 | 7.85 | 1136 |
| Acav_3650 | amino acid adenylation domain-containing protein | 1785 | 632 | 190795.1 | -0.043 | 6.02 | 1020 |
| Acav_3742 | methionyl-tRNA synthetase | 697 | 180 | 76841.4 | -0.208 | 5.61 | 0143 |
| Acav_3853 | surface antigen (D15) | 629 | 58 | 68306.4 | -0.427 | 9.19 | 0729 |
| Acav_3948 | UDP-N-acetylglucosamine pyrophosphorylase | 476 | 173 | 49441.2 | 0.007 | 6.71 | 1207 |
| Acav_4218 | AsnC family transcriptional regulator | 155 | 76 | 16696.3 | -0.041 | 9.35 | 1522 |
| Acav_4286 | flagellar basal body-associated protein FliL | 195 | 63 | 20959.0 | -0.292 | 7.92 | 1580 |
| Acav_4296 | flagellar hook-associated 2 domain-containing protein | 478 | 120 | 48675.0 | -0.070 | 7.70 | 1345 |
| Acav_4297 | Flagellin protein FlaB | 492 | 93 | 49291.9 | -0.096 | 5.74 | 1344 |
| Acav_4309 | flagellar biosynthetic protein FlhF | 533 | 106 | 57645 | -0.286 | 9.18 | 1419 |
| Acav_4446 | DNA-directed RNA polymerase subunit beta' | 4236 | 103 | 344174.4 | 0.922 | 4.69 | 0086 |
| Acav_4447 | DNA-directed RNA polymerase subunit beta | 4125 | 969 | 335465.6 | 0.934 | 4.70 | 0085 |
| Acav_4468 | leucyl-tRNA synthetase | 897 | 288 | 99852.6 | -0.322 | 5.85 | 0495 |
| Acav_4483 | phosphoribosylaminoimidazole carboxylase ATPase subuni | 393 | 58 | 41238.2 | 0.102 | 6.34 | 0026 |
| Acav_4497 | histidine kinase | 461 | 345 | 49148.9 | 0.025 | 5.56 | 0642 |

| | | | | | | | |
|---|---|---|---|---|---|---|---|
| Acav_4501 | pyruvate kinase | 478 | 997 | 51391.3 | -0.091 | 6.54 | 0469 |
| Acav_4502 | integral membrane sensor signal transduction histidine kina | 518 | 58 | 54817.9 | 0.033 | 9.85 | 0642 |
| Acav_4515 | transketolase | 695 | 664 | 74246.0 | -0.118 | 6.27 | 0021 |
| Acav_4522 | OmpA/MotB domain-containing protein | 296 | 97 | 31827.6 | -0.422 | 9.65 | 2885 |
| Acav_4556 | DNA topoisomerase III | 982 | 127 | 107873.9 | -0.456 | 9.05 | 0550 |
| Acav_4593 | (glutamate--ammonia-ligase) adenylyltransferase | 918 | 154 | 101692.3 | -0.294 | 6.35 | 1391 |
| Acav_4618 | Fimbrial protein pilin | 173 | 64 | 18024.6 | 0.030 | 8.67 | 2165 |
| Acav_4665 | UvrABC system protein A | 1017 | 139 | 111568.3 | -0.313 | 6.06 | 0178 |
| Acav_4752 | SurA domain-containing protein | 476 | 62 | 52397.1 | -0.463 | 6.76 | 0760 |
